# Critical evaluation of faecal microbiome preservation using metagenomic analysis

**DOI:** 10.1038/s43705-021-00014-2

**Published:** 2021-05-05

**Authors:** Alena L. Pribyl, Donovan H. Parks, Nicola Z. Angel, Joel A. Boyd, Alexander G. Hasson, Liang Fang, Samantha L. MacDonald, Blake A. Wills, David L. A. Wood, Lutz Krause, Gene W. Tyson, Philip Hugenholtz

**Affiliations:** 1 Microba Life Sciences, Brisbane, QLD Australia; 2 Centre for Microbiome Research, School of Biomedical Science, Translational Research Institute, Queensland University of Technology, Woolloongabba, QLD Australia; 3 Australian Centre for Ecogenomics, School of Chemistry and Molecular Biosciences, The University of Queensland, St Lucia, QLD Australia

**Keywords:** Metagenomics, Microbiome

## Abstract

The ability to preserve microbial communities in faecal samples is essential as increasing numbers of studies seek to use the gut microbiome to identify biomarkers of disease. Here we use shotgun metagenomics to rigorously evaluate the technical and compositional reproducibility of five room temperature (RT) microbial stabilisation methods compared to the best practice of flash-freezing. These methods included RNALater, OMNIGene-GUT, a dry BBL swab, LifeGuard, and a novel method for preserving faecal samples, a Copan FLOQSwab in an active drying tube (FLOQSwab-ADT). Each method was assessed using six replicate faecal samples from five participants, totalling 180 samples. The FLOQSwab-ADT performed best for both technical and compositional reproducibility, followed by RNAlater and OMNIgene-GUT. LifeGuard and the BBL swab had unpredictable outgrowth of *Escherichia* species in at least one replicate from each participant. We further evaluated the FLOQSwab-ADT in an additional 239 samples across 10 individuals after storage at −20 °C, RT, and 50 °C for four weeks compared to fresh controls. The FLOQSwab-ADT maintained its performance across all temperatures, indicating this method is an excellent alternative to existing RT stabilisation methods.

## Introduction

The last decade has seen a rapid increase in the number of faecal-based gut microbiome studies being conducted as an increasing number of links between the microbiome and diseases are uncovered.^1,2^ Key to all these studies is the ability to accurately preserve faecal samples until they can be transported to a laboratory for processing. If microbial constituents are compromised during this time, resulting analyses of the communities can produce false leads and inaccurate conclusions.^2^

Accepted best practice for preserving faecal samples is to immediately freeze at −20 to −80 °C,^3–5^ however this is typically not feasible for large-scale, geographically distributed studies. Room temperature (RT) preservation methods are therefore commonly used until samples can be frozen. These generally fall into the categories of a preservative solution, a dry swab or card. Most methods have been assessed for their ability to preserve microbial community structure using 16S rRNA gene sequencing.^3,5–16^ Although these studies find that inter-participant variability exceeds the variability introduced by the sample preservation method, changes in taxonomic composition amongst methods (within the same sample) are still detected^3,5,6,10,13–15,17^ (Supplementary Table S1). Using methods that minimise changes in taxonomic composition is essential for accurately measuring the microbiome and for identifying biomarkers related to a disease or intervention.

Gut microbiome studies are increasingly shifting to shotgun metagenomic sequencing because of the greater resolution metagenomics provides for both taxonomic and functional classification.^18–20^ To date, only a few studies have used metagenomics to assess RT preservation methods,^21–24^ and these studies primarily addressed compositional stability, but not technical (between-replicate) reproducibility. Here we use metagenomics to assess the technical and compositional (species and functional) reproducibility of five RT preservation methods compared to the conventional best practice of freezing. These included methods designed specifically for faecal sampling (OMNIgene-GUT™), more generic preservation (dry BBL CultureSwab™, RNAlater™), and methods designed for different sample types that have not been previously assessed with faecal samples (LifeGuard™—primarily for soil samples, FLOQSwab-ADT—primarily used for forensic DNA sampling). All methods were assessed using six replicate faecal samples from five participants. The FLOQSwab-ADT was the most reproducible of the methods tested and was further assessed with an additional ten participants at various storage temperatures to determine its range of use. To our knowledge, this is the most comprehensive study to assess both the compositional stability and technical reproducibility of this number of RT preservation methods with shotgun metagenomic sequencing.

## Methods

### Sample collection and storage—comparison of room temperature preservation methods

**Fig. 1:**
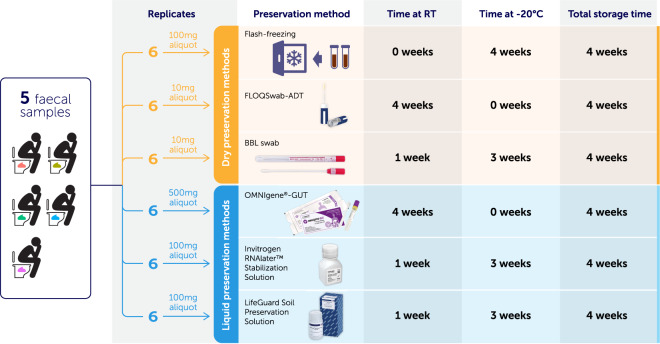
Study design for comparing five room temperature (RT) faecal stabilisation methods to a flash-frozen control. Faecal samples from five individuals were homogenised and a total of 36 aliquots were taken from each faecal sample. Six replicates were flash frozen, and six replicates were applied to each of the five RT preservation methods, for a total of 180 samples.

We tested five different RT preservation methods for their ability to accurately preserve microbial communities from faecal samples. These included 4N6 FLOQSwabs™ Genetics with an active drying tube (FLOQSwab-ADT; Product code 4504C; Copan Diagnostics, Murrieta, CA), OMNIgene-GUT™ (Catalogue no. OM-200; DNA Genotek Inc., Ottawa, CA), RNAlater™ (Catalogue no. AM7021; Thermo Fisher Scientific, Waltham, MA), LifeGuard™ Soil Preservation Solution (Catalogue no. 12868-1000; QIAGEN, Hilden, DE), and a dry BD BBL CultureSwab™ (BBL swab; Catalogue no. 220144; Becton, Dickinson and Company, Sparks, MD). The FLOQSwab-ADT and BBL swab are both dry collection methods whereas OMNIgene-GUT, RNAlater and LifeGuard are liquid-based preservatives. Each preservation method was assessed according to the manufacturer’s instructions for maximum storage time at room temperature, and if less than four weeks, was subsequently frozen at −20 °C until the end of four weeks (Fig. 1).

Whole faecal samples were collected on-site at the Translational Research Institute (Woolloongabba, QLD, AU) from five participants with no major medical conditions and delivered to the laboratory (within five minutes) for immediate processing. All participants provided informed consent for the use of their de-identified samples to be used for research purposes. A minimum of 12 g of the total faecal sample was placed into a sterile 50 ml collection container. The faeces were mixed with a sterile spatula for a minimum of 2 min to homogenise. Following homogenisation, the following amounts of faecal material were aliquoted to each of the preservation methods: (1) six, ~100 mg aliquots transferred to 2 ml Eppendorf tubes on dry ice and placed into a −20 °C freezer for four weeks; (2) six, ~10 mg aliquots applied to FLOQSwab-ADTs (FLOQSwabs are made of short nylon fibres arranged perpendicularly that quickly absorb the faecal material; faeces are subsequently desiccated on the swab when placed in the active drying tube) and stored at room temperature for four weeks; (3) six, ~500 mg aliquots transferred to OMNIgene-GUT sampling tubes, which were shaken according to the manufacturer’s instructions, and stored at room temperature for four weeks; (4) six, ~100 mg aliquots transferred to 2 ml Eppendorf tubes containing 1 ml of RNAlater, vortexed to reduce faecal clumping, stored at room temperature for one week, and then transferred to −20 °C for the remaining 3 weeks; (5) six, ~100 mg aliquots transferred to 2 ml Eppendorf tubes containing 1 ml of LifeGuard and stored at room temperature for one week, and then transferred to −20 °C for the remaining three weeks; and (6) six, ~10 mg aliquots applied to BBL swabs and stored at room temperature for one week and then transferred to −20 °C for the remaining three weeks (Fig. 1). This totalled 180 samples (five individuals × six replicates × six preservation methods).

### Sample collection and storage—comparison of FLOQSwab-ADT at different temperatures

Whole faecal samples were collected as before from ten participants (see above), and ~10 mg aliquots were transferred to: (1) six FLOQSwab-ADTs and immediately extracted, (2) six FLOQSwab-ADTs and immediately transferred to −20 °C for four weeks, (3) six FLOQSwab-ADTs and stored at room temperature for four weeks, and (4) six FLOQSwab-ADTs and stored at 50 °C for four weeks. This totalled 240 samples.

### DNA extraction and metagenomic sequencing

Total DNA was extracted from faecal samples with the QIAamp 96 PowerFecal QIAcube HT Kit using the QIAcube HT DNA extraction system (QIAGEN, Hilden, DE). Manufacturer’s instructions were followed, except for a modification in the initial mechanical lysis to include a bead-based lysis with 0.1 mm glass beads in a 2 ml deep well plate for 10 min. DNA was eluted into a total volume of 100 μl. Total DNA was quantified using the QuantIT HS Kit (Thermo Fisher Scientific, Waltham, MA). The extracted DNA was processed using the Nextera-XT Library preparation Kit (Illumina, Inc., San Diego, CA) with v2 Index sets A–D (Illumina, Inc., San Diego, CA). Indexed libraries with an average insert size of 500 bp were pooled equally and sequenced at a target depth of 7 M reads per sample on the NovaSeq 6000, using a 2 × 150 bp paired end v1 sequencing kit. Positive (mock communities) and negative (reagent only) controls were included in the DNA extraction and library preparation protocols to confirm appropriate and consistent processing was achieved. The positive DNA extraction controls included: (1) a 100 mg aliquot from a homogenised donor faecal sample (same sample repeated on every run), (2) ZymoBIOMICS Microbial Community Standard (Catalogue # D6300; Zymo Research, Irvine, CA), and (3) ZymoBIOMICS Gut Microbiome Standard (Catalogue # D6331; Zymo Research, Irvine, CA). The positive library preparation controls included: (1) extracted DNA from a donor faecal sample (same DNA sample repeated on each run), (2) 20 Strain Even Mix Genomic Material (ATCC^®^ MSA-1002™; Manassas, Virginia), and (3) ZymoBIOMICS Microbial Community DNA Standard (Catalogue # D63056; Zymo Research, Irvine, CA). Sequencing runs were passed if positive controls had an average Hellinger transformed beta diversity distance of ≤0.2 compared to the ‘expected’ compositions.

### Quality control of sequencing reads

Paired reads from each sample were processed to remove potential human contamination by mapping reads to the human reference genome (GRCh38.p12) using the MEM method of BWA v0.7.17-r1188.^25^ Read pairs with the expected orientation where either read had a percent identity ≥95% and percent alignment length ≥90% were considered human contamination and removed from the sample. Remaining reads were processed using Trimmomatic v0.36^26^ to remove adaptors, filter leading or trailing bases with a quality score <3, clip reads when the average 4-base window had a quality score <15, and discard reads <100 bp in length after applying the previous QC steps. Samples were than randomly subsampled to 7 M paired reads using seqtk v1.2-r94 (https://github.com/lh3/seqtk) to account for differences in sequencing depth. Sequencing depths for the first experiment comparing preservation methods ranged from 10.9 to 31.2 M paired reads (3.3–9.4 Gbp), with a mean of 19.5 M paired reads (5.9 Gbp). Sequencing depths for the second experiment comparing temperature treatments of the FLOQSwab-ADT ranged from 6.2 M to 19.4 M paired reads (1.9–5.8 Gbp), with a mean depth of 10.7 M paired reads (3.2 Gbp). One of the six replicates from Participant 9 in the second experiment failed quality control and was removed from the analysis. In addition, two of the remaining 239 samples from the second experiment fell below 7 M reads, at 6.2 and 6.8 M reads. These two samples were still included in the analysis.

### Exploring community composition

Species profiles were obtained using the commercially available Microba Community Profiler (MCP) v2.0.0 using the Microba Genome Database (MGDB) v1 as a reference database (Microba Life Sciences, Brisbane, Australia; https://microba.com/microbiome-research).^27^ The MGDB v1 uses the Genome Taxonomy Database (GTDB) Release 02-RS83 taxonomy and consists of an expanded set of genomes to those in the GTDB. Species profiles were also obtained using the publicly available MetaPhlAn3, which was run with the parameter ‘–unknown _estimation’.^28^

Functional profiles were obtained by first annotating protein coding genes from MGDB v1 genomes with Enzyme Commission ids by mapping to the UniRef90 database^29^ using 90% amino acid identity, over 80% of the query and subject sequence. Each genome was then annotated with pathways from the MetaCyc Metabolic Pathway Database v22.6 where >80% of the enzymes were encoded by the genome.^30^ Functional profiles were calculated by aggregating the abundance of genomes annotated with each pathway.

Bray-Curtis dissimilarity was calculated using R with the vegan package v.2.5-6.^31^ Weighted UniFrac (W-UniFrac) distances of relative abundances was calculated using R with the phyloseq package v1.32-0.^32^ W-UniFrac distances were calculated for MCP derived profiles using a reference tree spanning the 23,458 bacterial species representatives in GTDB Release 04-RS89 along with 3,517 new bacterial species representatives in the MGDB. The tree was inferred with FastTree 2.1.11 using the WAG+GAMMA model and a concatenated MSA of the 120 bacterial marker genes used by the GTDB.^33^ Identification and alignment of these marker genes was done with GTDB-Tk v1.3.0.^34^ For technical reproducibility, Bray–Curtis dissimilarity and W-UniFrac distances were calculated between replicates for each participant and preservation method. For compositional stability, Bray–Curtis dissimilarity and W-UniFrac distances were calculated between each preservation method and the control samples. Principal components analyses were based on Hellinger-transformed species and functional relative abundances to reduce outlier effects. Box-and-whisker, stacked bar and principal component analysis plots were generated with the R package ggplot2 v3.3.2.^35^ The box-and-whisker plot shows the lower and upper quartiles as a box, the median value as a line within the box, 1.5 times the interquartile range as whiskers, and outliers as crosses. Shannon diversity (*H*’ = −Σ_pi_ ln_pi_, where _pi_ is the relative abundance of the *i*th species), richness (*R* = number of species or the number of pathways >80% complete present in a sample), and Shannon or Pielou evenness (*J’* = *H’/lnR*) were calculated using the R package vegan v2.5-6.^31^

### Statistical analyses

All statistical analyses were calculated using R, v3.5.1. *P* values were corrected for multiple testing by False Discovery Rate (FDR), where an FDR of <0.05 was considered significant.

Differences between technical replicates were assessed using Bray-Curtis dissimilarity and W-UniFrac distances. Differences in inter-replicate Bray–Curtis dissimilarity or W-UniFrac distances of each RT method were compared to control samples using linear mixed-effects regression (LMER), modelling Bray–Curtis dissimilarity or W-UniFrac distances as the dependent variable, preservation method as fixed effect and participant as random effect. The amount of inter-replicate variance attributed to the participant was assessed using permutational multivariate analysis of variance (PERMANOVA) of dissimilarities, where permutations were set to 10^4^. PERMANOVA analyses were run using the Adonis function in R.

Compositional reproducibility of profiles was assessed using Shannon diversity, evenness, richness, Bray–Curtis dissimilarity and W-UniFrac distances compared to control samples. Differences between preservation methods or temperature treatments were assessed using LMER, modelling either alpha or beta diversity measures as the dependent variable, preservation method as a fixed effect and participant as a random effect. The source of the variance for each method or treatment was assessed using Adonis PERMANOVA, as above. To determine differences between the relative abundance of individual species from each preservation method compared to frozen controls, centre log ratio transformed relative abundances were compared using LMER.

## Results

### Comparison of room temperature preservation methods

In this experiment, five methods were investigated for their ability to preserve the microbial communities of faecal samples at RT from five participants, compared to flash-frozen controls. Given conventional requirements for storing faecal samples for long periods before processing, preserved samples were stored for the maximum recommended time at RT (one or four weeks; according to manufacturer’s instructions) prior to freezing. Six replicate samples from each participant and preservation method were sequenced, totalling 180 samples.

### Technical reproducibility of different preservation methods

**Fig. 2:**
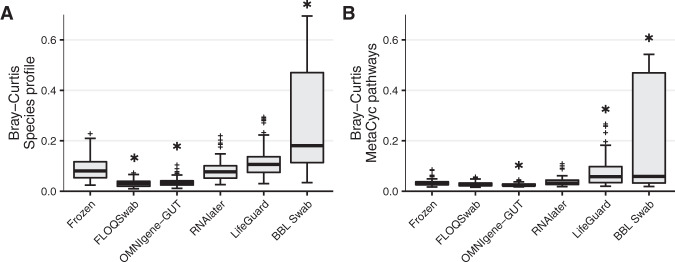
Between replicate technical variability of species and functional profiles for each stabilisation method. **A** Aggregated Bray-Curtis dissimilarity between replicate species profiles of all participants for each of the stabilisation methods. **B** Aggregated Bray-Curtis dissimilarity between replicate functional profiles of all participants for each of the stabilisation methods. * = FDR *P* value < 0.05 compared to frozen samples. Significance was assessed by linear mixed effect regression (LMER).

Technical (between replicate) reproducibility was assessed for both microbial species and functional profiles for each preservation method (Fig. 2, Supplementary Figs. S1–S3). For species profiles, BBL swab had significantly higher technical variability compared to frozen controls, and FLOQSwab and OMNIgene-GUT had the lowest amount of technical variance (Fig. 2A, Supplementary Fig. S2). Adonis PERMANOVA analysis and W-UniFrac analyses confirmed these results (Supplementary Fig. S3). Functional profiles generally had less technical variability than species profiles (Fig. 2B). Despite this, BBL swab and LifeGuard had significantly higher technical variability of functional profiles compared to frozen samples while OMNIgene-GUT had the lowest variability. Again, Adonis PERMANOVA analysis confirmed these results, with FLOQSwab-ADT, OMNIgene-GUT and RNAlater having the lowest and BBL swab having the highest residual variance (Supplementary Fig. S3).

### Impact of preservation methods on species and functional profiles

Participants accounted for the majority of variance associated with species profiles (ranging from 64% variance for BBL swab to 84% variance for FLOQSwab-ADT) and functional profiles (ranging from 51% variance for BBL swab to 90% variance for RNAlater) in all five preservation methods (Supplementary Figs. S4–S6). However, comparisons of alpha and beta diversity revealed substantial differences in the ability of the methods to preserve species and functional profiles relative to flash-frozen controls.

**Fig. 3:**
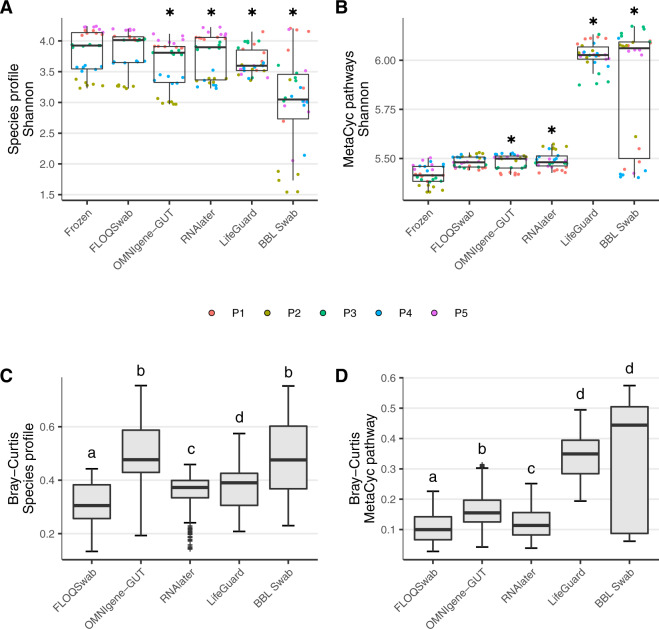
Compositional reproducibility of species and functional profiles. **A** Aggregated Shannon diversity based on species profiles across all participants for each storage method. **B** Aggregated Shannon diversity based on functional profiles across all participants for each storage method. **C** Aggregated Bray–Curtis dissimilarity of species profiles from all participants for each stabilisation method. **D** Aggregated Bray-Curtis dissimilarity of functional profiles from all participants for each stabilisation method. Significance was assessed by linear mixed effect regression (LMER). **A**, **B** Different colours represent different participants. * = FDR *P* value < 0.05 compared to frozen samples. **C**, **D** Boxes that do not share the same letter are significantly different at FDR *P* value < 0.05.

FLOQSwab-ADT, OMNIgene-GUT and RNAlater had similar Shannon diversity, evenness and richness compared to the flash-frozen samples for species profiles derived from the MCP, and LifeGuard also had similar species richness. In contrast, BBL swab and to a lesser extent, LifeGuard, had substantially reduced Shannon diversity and evenness of species profiles (Fig. 3A, Supplementary Fig. S7). When species profiles were derived from MetaPhlAn3, only OMNIgene-GUT and the BBL swab had significantly reduced Shannon diversity and evenness compared to the flash-frozen control (Supplementary Fig. S8). Functional profiles showed slightly increased Shannon diversity and evenness for FLOQSwab-ADT, OMNIgene-GUT and RNAlater, and substantially increased diversity, evenness and richness for LifeGuard and BBL swab (Fig. 3B, Supplementary Fig. S7).

**Fig. 4:**
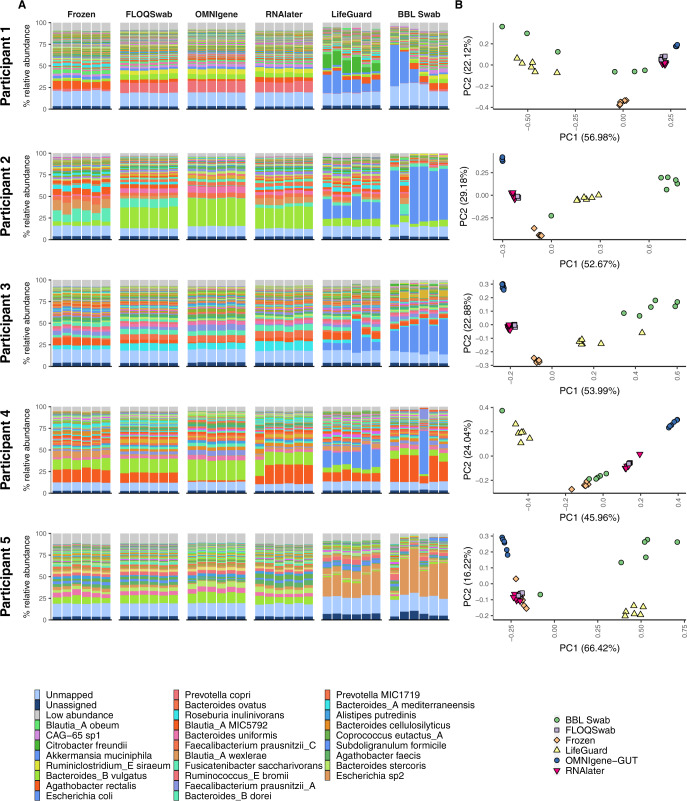
Comparison of species profiles for each replicate from the five participants (Fig. 1). Profiles are organised by participant and stabilisation method. **A** Bar chart of microbial species with the highest mean abundance for each participant. The light and dark blue bars at the bottom of each bar plot indicate the percentage of unmapped and unassigned reads, respectively. The light grey bar at the top of each bar plot indicates the proportion of species with a minimum abundance <0.5% across all samples from a participant. **B** Principal component analysis plots of Hellinger transformed species profiles are provided for each participant with each stabilisation method depicted by a different colour and shape combination.

Bray-Curtis dissimilarity and W-UniFrac distances of each method compared to flash-frozen controls indicated FLOQSwab-ADT and RNAlater had the most similar species profiles to the flash-frozen control. Species profiles from OMNIgene-GUT and BBL swab varied the most relative to flash-frozen samples (Fig. 3C, Supplementary Figs. S6a, S9a, S10d). This result was replicated when species profiles were derived from MetaPhlAn3 (Supplementary Fig. S10b). Functionally, the FLOQSwab-ADT, RNAlater and OMNIgene-GUT had the most similar profiles to flash-frozen samples, whereas LifeGuard and BBL swab profiles diverged the most (Fig. 3D, Supplementary Figs. S6, S9b). These results were consistent with PCA plots for each participant where species and functional profiles from LifeGuard and BBL swab tended to cluster separately from the other methods (Fig. 4B, Supplementary Figs. S11b, S12b).

The most notable difference in species profiles was attributed to a large and unpredictable outgrowth of *Escherichia coli* or *Escherichia sp2* in BBL swab and LifeGuard (Fig. 4A, Supplementary Fig. S13, Supplementary Table S2). LifeGuard also had a large outgrowth of *Citrobacter freundii* in Participant 1. These species were below 0.05% relative abundance in the flash-frozen samples and likely explain the differences in alpha diversity for both species and functional profiles when compared to flash-frozen samples (Fig. 3A, B). Specifically, the high relative abundance of *Escherichia spp*. reduced species-level Shannon diversity whereas the additional metabolic pathways present in these facultative anaerobes increased the functional Shannon diversity. For example, there was an increase in the relative abundance of peptidoglycan maturation and lysine degradation in BBL swabs and LifeGuard compared to flash-frozen samples (Supplementary Fig. S11a), which we attribute to the increased relative abundance of *Escherichia* species (Fig. 4A).

Species and functional variation compared to flash-frozen samples was significantly less in FLOQ Swab-ADT and RNAlater than in BBL swab and LifeGuard (Fig. 3C, D). Numerous small, but significant changes in species relative abundances were consistent between FLOQSwab-ADT, RNAlater and OMNIgene-GUT relative to the flash-frozen samples, although the largest magnitude of change was observed in OMNIgene-GUT samples (Supplementary Fig. 4A, Supplementary Fig. S13, Supplementary Table S2). These included an increased relative abundance of *Bacteroides_B vulgatus, Bacteroides uniformis, Prevotella copri, and Ruminiclostridium siraeum* and a decreased abundance of *Agathobacter rectalis* and *Blautia_A wexlerae*. In addition, there were some changes in OMNIgene-GUT profiles relative to flash-frozen samples that were not observed in the other methods, such as an increased relative abundance of *Faecalibacterium prausnitzii_A and Faecalibacterium prausnitzii_B*, and decreased *Ruminococcus_E bromii*, and *Fusicatenibacter saccharivorans*. Relative abundance of functional pathways for FLOQSwab-ADT, RNAlater and OMNIgene-GUT relative to the flash-frozen samples showed less variation than species relative abundances (Supplementary Fig. S11).

These results indicate that for both technical reproducibility and compositional stability of faecal samples stored at RT, the FLOQSwab-ADT performs best, followed closely by RNAlater and then by OMNIgene-GUT. LifeGuard and BBL swab were the least reproducible due to outgrowth of facultatively anaerobic species.

### Stability of the FLOQSwab-ADT profiles at different temperatures

We next investigated the top ranked RT method, which utilises the FLOQSwab-ADT, to preserve faecal microbial communities across a range of storage temperatures. To minimise variation in the control samples, we used freshly collected and extracted samples. Faecal samples were collected from ten participants and either processed immediately as fresh samples or stored at −20 °C, RT, and 50 °C for a period of four weeks. Six replicate samples were assessed from each participant and temperature treatment for a total of 240 samples. However, one sample from Participant 9 failed quality control and was removed, thus only 239 metagenomes were used in the final analysis. As with the previous experiment, metagenomic sequencing was performed on all samples from which species and functional profiles were generated.

**Fig. 5:**
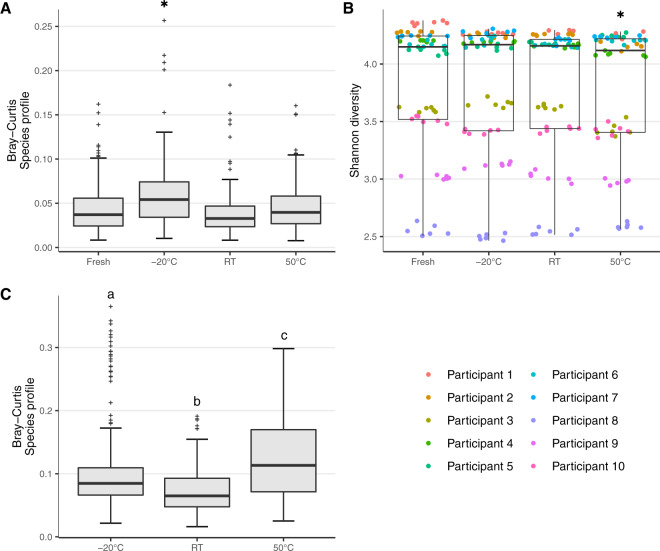
Technical and compositional reproducibility of each temperature treatment compared to fresh controls. **A** Technical reproducibility assessed using aggregated Bray-Curtis dissimilarity of replicate species profiles from all participants, at each temperature treatment. **B** Compositional reproducibility assessed using aggregated Shannon diversity of species profiles for all participants at each temperature treatment. **C** Compositional reproducibility assessed using aggregated Bray-Curtis dissimilarity of species profiles for each temperature treatment compared to fresh controls. Statistical significance was assessed by LMER. * = FDR *P*-value < 0.05 compared to fresh samples. Different colours represent different participants. Grey crosses represent outliers. Letters: Boxes that do not share the same letter are significantly different at FDR *P* value < 0.05.

There was no difference in technical reproducibility of species profiles derived from the MCP between fresh samples, RT and 50 °C treatments (Fig. 5A). Only the −20 °C treatment had a significantly increased Bray–Curtis dissimilarity between replicate species profiles compared to fresh samples, although the difference in participant variance between fresh and −20 °C treated swabs was less than 0.5% (Supplementary Fig. S14). There were no differences in technical reproducibility when species profiles were analysed using W-UniFrac or when species profiles were derived from MetaPhlAn3 (Supplementary Fig. S15a, c). In addition, no significant differences in technical reproducibility were observed when comparing functional profiles across the different temperature treatments to fresh profiles and the maximum difference in participant variance between fresh and temperature treated functional profiles was 1.6% (Supplementary Figs. S14, S16a).

Species stability assessed using alpha diversity showed a small but significant reduction in Shannon diversity and evenness in the 50 °C treatment, and small but significant increases in species richness across all temperature treatments compared to fresh samples (Fig. 5B, Supplementary Fig. S17). No differences in Shannon diversity, richness and evenness were observed when species profiles were derived from MetaPhlAn3 (Supplementary Fig. S18). Functional profiles showed small, but significant reductions in Shannon diversity and evenness in the −20 °C treatment compared to the fresh samples and both −20 °C and 50 °C treatments had small but significant increases in functional richness (Supplementary Fig. S16b–d).

Bray-Curtis dissimilarity of species and functional profiles compared to the fresh samples also showed small, but significant differences between the temperature treatments (Fig. 5C, Supplementary Figs. S15b, S16e). The RT species profiles were closest to fresh profiles while the 50 °C profiles were the most different (Fig. 5C). W-UniFrac distances of species profiles showed no differences between the temperature treatments (Supplementary Fig. S15d). Functional profiles also showed RT profiles were closest to fresh profiles (Supplementary Fig. S16e). These results were confirmed with Adonis PERMANOVA, but showed that the difference between treatments was minimal, with the amount of variance explained by other sources comprising a maximum of 2.3% in species profiles and a maximum of 7.7% in functional profiles (Supplementary Fig. S19).

**Fig. 6:**
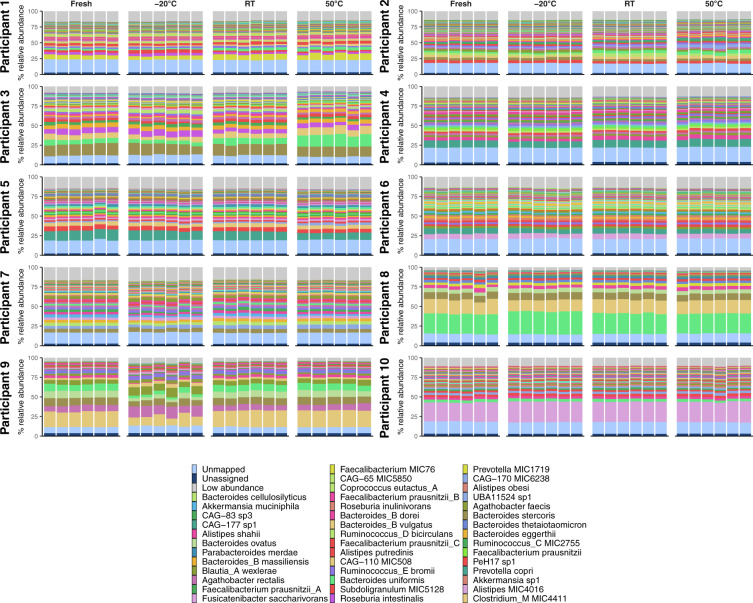
Comparison of species profiles for the 239 samples from the ten participants. Profiles are organised by participant and treatment method. The bar charts list the ten species with the highest mean abundance for each participant. The light and dark blue bars at the bottom of each bar plot indicate the percentage of unmapped and unassigned reads, respectively. The light grey bar at the top of each bar plot indicates the proportion of species with a minimum abundance <0.5% across all samples from a participant.

As in the previous experiment, the great majority of variance is attributed to the participant (Supplementary Fig. S19), which can also be observed in taxonomic PCA plots that show a clear separation between participant profiles and a high degree of overlap between temperature treatments for a given participant (Supplementary Figs. S20, S21). Bar plots of species and functional profiles for each participant and temperature treatment reveal minimal shifts in community composition, regardless of the treatment (Fig. 6, Supplementary Figs. S22, S23). This suggests that FLOQSwab-ADT robustly preserves both the species and functional profiles of the original sample at −20 °C, RT and 50 °C for at least 4 weeks.

## Discussion

As the study of the gut microbiome progresses from disease associations to the identification of biomarkers and mechanisms of action, it is essential that the methods used to collect faecal samples can produce community profiles as close as possible to those of freshly collected samples. In this study, we rigorously evaluated five RT preservation methods for both technical reproducibility and compositional stability using shotgun metagenomics. These included two of the most commonly used methods in faecal microbiome studies, OMNIgene-GUT^3,6,36^ and RNAlater.^3,6,23^ We also included the dry BBL CultureSwab used in the citizen science AmericanGut Project^37^ and LifeGuard as a widely used, but more generic RT preservation method for environmental samples.^38–40^ Lastly, we included the FLOQSwab, which has been used in faecal microbiome studies,^41–44^ but not in combination with an active drying tube (ADT). FLOQSwab-ADT was specifically designed for the preservation of forensic samples.^45^

OMNIgene-GUT and FLOQSwab-ADT had the highest technical reproducibility of the preservation methods tested. However, it was the FLOQSwab-ADT and RNAlater that were best able to preserve species and functional profiles, respectively, compared to freezing. This confirms previous findings that OMNIgene-GUT has high technical reproducibility,^3,6^ although conflicts with reports that OMNIgene-GUT has higher compositional stability compared to RNAlater.^3,6,13,21,46,47^ One possible reason for the discrepancy is that we stored samples with OMNIgene-GUT at RT for 4 weeks, whereas several other studies have used shorter time periods ranging from 24 h to two weeks.^6,13,46,47^ In addition, studies assessing RNAlater have had mixed results with some finding high compositional stability^3,7,12,22,24^ and others finding the opposite.^5,6,8^ These conflicting results may be due to difficulties in evenly dispersing faecal samples in RNAlater solution during collection^8^ and difficulties in separating RNAlater from faeces during DNA extraction.^5,43^ We took particular care to reduce clumping of faecal samples in RNAlater and did not experience difficulties in separating RNAlater from faeces during DNA extraction, which may explain the strong performance of this method in preserving community profiles.

The BBL swab and LifeGuard storage methods showed varying levels of outgrowth from facultative anaerobic organisms such as *Escherichia* species and *Citrobacter freundii*, despite these species being below 0.05% relative abundance in the flash-frozen baseline samples. Outgrowths of facultative anaerobic species from the family Enterobacteriaceae when using a dry swab for sample collection has been previously observed,^15,17^ and the American Gut Project has even developed an algorithm to bioinformatically correct for outgrowth on a dry swab.^17^ However, to our knowledge this is the first report of the growth of facultatively anaerobic species in LifeGuard, which was designed as an RNA preservative for microbial communities from soil, but has been applied to both DNA and RNA from diverse environmental samples.^38–40^ Our results indicate that BBL swabs and LifeGuard are unable to prevent growth of facultative anaerobes during storage of faecal samples at RT for one week. For the BBL swab this is likely due to oxygen exposure and availability of nutrients in the faecal material. The cause of the outgrowth in LifeGuard may be attributable to nutrients present in the residual faecal material, or possibly nutrients in LifeGuard itself as we found *E. coli* could grow in the unamended reagent (Supplementary information**-1**). Outgrowth of facultative anaerobes is particularly concerning for studies that are attempting to identify biomarkers for disease and therapeutic targets, as they may cause researchers to pursue false leads. We strongly recommend against the use of dry BBL swabs and LifeGuard for RT storage of faecal samples and caution against the use of microbiome data derived from this method for the identification of biomarkers.

The FLOQSwab-ADT has not been previously evaluated for the preservation of faecal samples, as it was designed for the preservation of human DNA. Studies have assessed FLOQSwabs dry, and in combination with liquid preservation solutions, but not in combination with the ADT.^41–44^ The FLOQSwab is designed to absorb and release sample material more effectively than traditional fibre swabs, while the ADT is designed to quickly dry the specimen to optimise DNA stabilisation.^48^ FLOQSwab-ADT was included in our study because we were interested in a non-liquid method for preserving faecal samples at RT, and previous studies have shown that desiccation of faecal samples on cards can produce reliable results.^3,7,9^ The FLOQSwab-ADT performed amongst the best for both technical and compositional reproducibility (Figs. 2, 3). Further testing of the FLOQSwab-ADT at various temperatures compared to fresh samples demonstrated that it performed best at RT, however still preserved the microbial community with high technical and compositional reproducibility at −20 and 50 °C for 4 weeks. This suggests that samples can be transported through the post year-round and seasonal fluctuations in temperature will not compromise the sample.

Interestingly, the species shifts observed in the FLOQSwab-ADT in the first experiment relative to the frozen control, such as an increased relative abundance of *Bacteroides spp*. and *Prevotella copri* and decreased abundance of *Agathobacter rectalis*, were not observed in the second experiment when the comparison was to fresh samples rather than frozen. This suggests that freezing faecal samples with no preservative at −20 °C for a period of 4 weeks may change the microbial community structure and that the species shifts we observed in the first experiment between the FLOQSwab-ADT, RNAlater and OMNIgene-GUT and flash-frozen samples may have been a result of the −20 °C treatment altering the community rather than the preservation methods. Only a couple of studies have assessed taxa-level differences between fresh and fresh-frozen samples;^3,49^ these studies observed trends towards decreased levels of the genera *Bacteroides* and/or *Prevotella* in frozen samples relative to fresh samples, consistent with our findings. Additionally, a study investigating the effect of long-term storage of faecal samples at −80 °C for use in faecal microbiota transfers observed a trend of decreasing levels of *Bacteroides spp.* with longer storage, with differences becoming significant at 3 months.^50^ These results suggest that the current best practice of freezing samples without stabilisation may result in changes to specific taxa, in particular species from the *Bacteroides* genus. Further study, preferably using shotgun metagenomics, should be conducted to determine if freezing faecal samples without stabilisation results in changes to specific microbial species and if freezing should still be considered a ‘best practice.’

In conclusion, the FLOQSwab-ADT and RNAlater had the best combination of technical reproducibility and microbial community preservation, with OMNIgene-GUT also performing acceptably well. All three methods, therefore, provide viable options for preserving faecal samples at RT. However, it should be noted that RNAlater samples can only be stored at RT for 1 week according to manufacturer’s instructions and faecal clumping can be an issue, which may limit the application of this method in certain circumstances. The FLOQSwab-ADT is the most versatile method, as it does not use a liquid preservation solution and accurately reproduces the microbial species and functional profiles of faecal samples at temperatures up to 50 °C for a period of 4 weeks. This makes the FLOQSwab-ADT an excellent option for use in direct to consumer and research-focused faecal sample preservation, especially where faecal samples need to be collected and transported across long distances or sent through the post.

## Supplementary information

Supplementary Information

Supplementary Table S1

Supplementay Table S2

Supplementary Table S3

## Data Availability

The data for this study have been deposited in the European Nucleotide Archive (ENA) at EMBL-EBI under accession number PRJEB41481. Metadata for the samples are in Supplementary Table S3. All additional data relevant to the study are included in the article or uploaded as supplementary information.
