# Supplementary material for: Critical evaluation of faecal microbiome preservation using metagenomic analysis
Source: ISME Commun. 2021 May 5;1:14. doi: 10.1038/s43705-021-00014-2 (PMC9645250; doi:10.1038/s43705-021-00014-2)
Supplement: Supplementary file 1 — Supplementary Information [file 43705_2021_14_MOESM1_ESM.docx]

**Supplementary Information**

**SI-1. Confirmation of growth of facultative anaerobes in LifeGuard soil preservation solution**.

**Methods**

One strain of *Citrobacter freundii* and one strain of *Escherichia coli* were sourced from the Microba Biobank and used to determine their growth potential in LifeGuard. Each bacterial strain was combined with 2 ml LifeGuard in a 15ml Falcon tube, in triplicate. We additionally had one treatment of *C. freundii* in LifeGuard where a 100mg aliquot of the original faecal sample from Participant 1 (which had the original outgrowth of *C. freundii*) was added.

1. 3 replicates: 1 x 10^^8^ cells of *E. coli* + 2ml LifeGuard solution
2. 3 replicates: 1 x 10^^8^ cells of *C. freundii* + 2ml LifeGuard solution
3. 3 replicates: 1 x 10^^8^ cells of *C. freundii* + 2ml LifeGuard solution + 100mg aliquot faecal sample

All samples were stored at room temperature (RT; without shaking) for 7 days and subsequently assessed at Days 0 and Day 7 for evidence of bacterial growth (18 samples total). In samples incubated in LifeGuard alone, cell numbers were assessed using the Femto DNA quantification kit (Catalog # E2006; Zymo Research, Irvine, CA) according to the manufacturer’s instructions, and data reported as a cell number. Due to the presence of other microorganisms, the samples containing faecal material were assessed by metagenomic sequencing as described in the Methods section, and data is reported as a relative abundance of *C. freundii*. Growth of *C. freundii* and *E. coli* was assessed by calculating the fold change (FC) in either cell count or relative abundance from Day 0 to Day 7.

**Results and Discussion**

*E. coli* increased in abundance when incubated at RT for 7 days in LifeGuard alone, however *C. freundii* did not (**Table S4**). When faecal material was added however, growth of *C. freundii* did occur. This demonstrates that LifeGuard is unable to prevent the growth of the facultative anaerobes *E. coli* and *C. freundii*. In fact, LifeGuard alone appears to support the growth of *E. coli*, suggesting that *E. coli* can use it as a nutrient source. By contrast, *C. freundii* requires an additional nutrient source for growth in LifeGuard.

**SUPPLEMENTARY TABLES**

**Supplementary Table S1.** Excel file. Overview of treatment-control comparison studies that assess efficacy of OmniGene-GUT, RNAlater and BBL swab/sterile swab for room temperature (RT) storage of stool samples. Studies were only included if samples were stored at RT for a minimum of 2 days.

**Supplementary Table S****2.** Excel file. Comparison of species relative abundances for each preservation method compared to flash-frozen controls. Relative abundances were centre-log ratio transformed and statistical significance was assessed using linear mixed effects regression (LMER).

**Supplementary Table S3.** Excel file. Associated metadata for metagenomes deposited in the European Nucleotide Archive.

**Supplementary Table S4.** Fold change of *C. freundii* or *E. coli* when stored in LifeGuard, with or without a nutrient source, at room temperature for 7 days.

| Organism | Stabilisation solution | Nutrient source | Mean Day 0 (±stdev) | Mean Day 7 (±stdev) | Fold change |
| --- | --- | --- | --- | --- | --- |
| *E. coli* | LifeGuard | None | 1.81 x 10^8^ cells (±2.94 x 10^6^) | 1.25 x 10^9^ cells (±2.62 x 10^7^) | 7 |
| *C. freundii* | LifeGuard | None | 1.81 x 10^8^ cells (±5.44 x 10^6^) | 1.48 x 10^8^ cells (±3.40 x 10^6^) | <0 |
| *C. freundii* | LifeGuard | Faeces | 3.35% (±0.66%) | 24.49% (±5.16%) | 7 |

**SUPPLEMENTARY FIGURES**


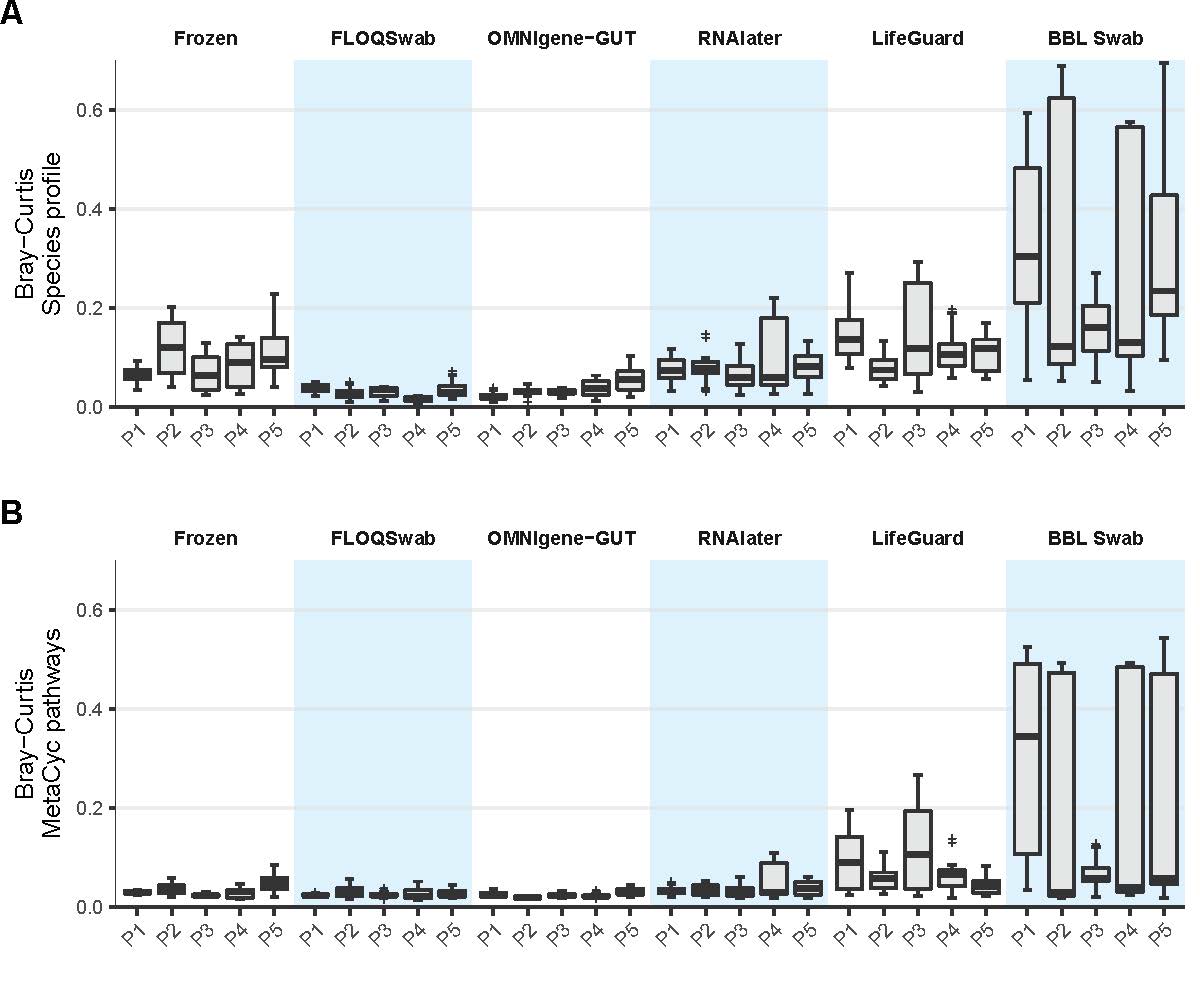


**Supplementary Figure S1**. Technical reproducibility of species (MCP) and functional profiles by participant, assessed using Bray-Curtis dissimilarity. **(A)** Bray-Curtis dissimilarity between replicates for species profiles by participant. **(B)** Bray-Curtis dissimilarity between replicates for species profiles by participant.


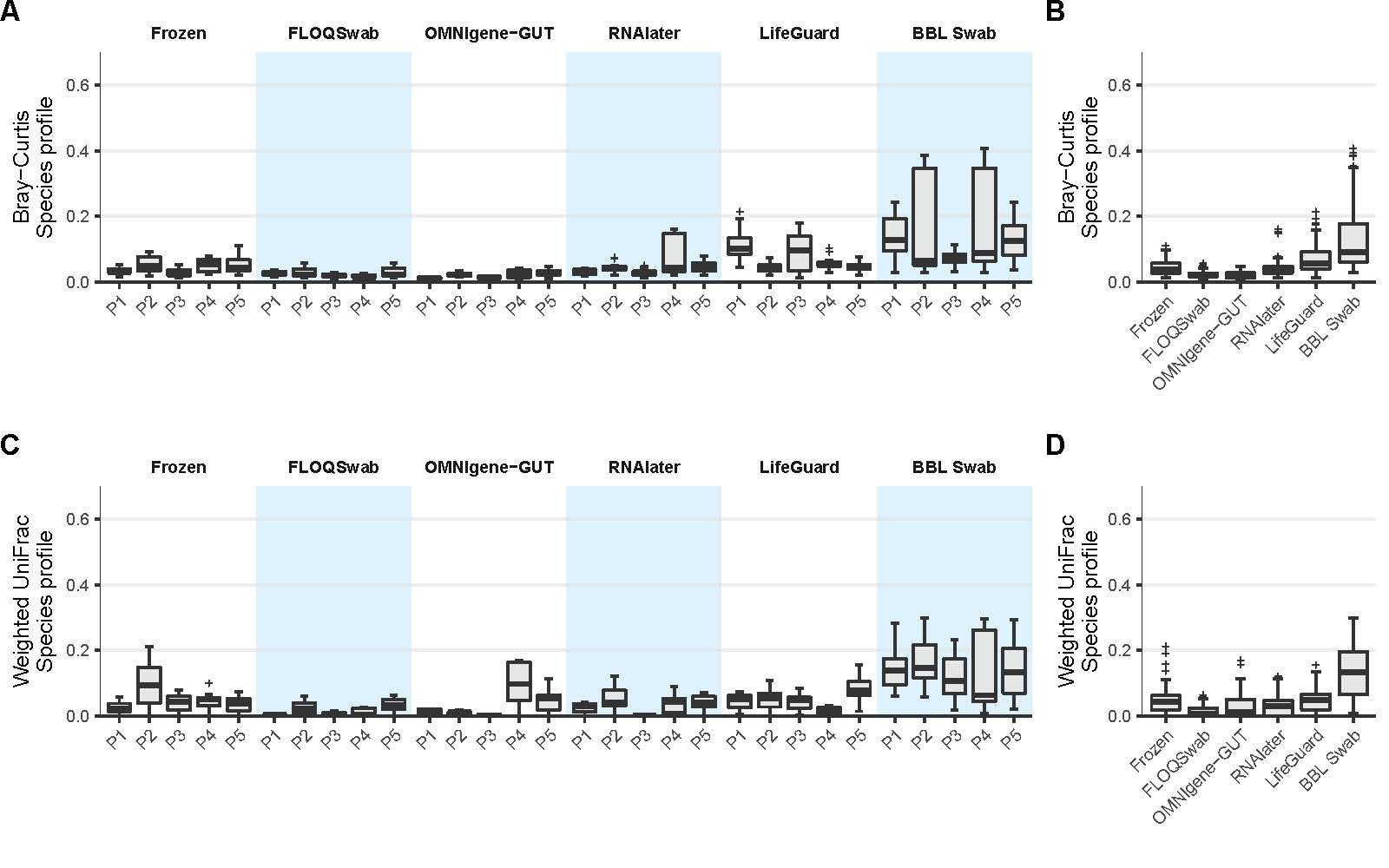


*

*

*

*

*

*

**Supplementary Figure S2**. Technical variability of species profiles for each stabilisation method. **(A)** Bray-Curtis dissimilarity between replicate species profiles derived from **MetaPhlan3**, by participant for each stabilisation method. **(B)** Aggregated Bray-Curtis dissimilarity between replicate species profiles derived from **MetaPhlan3**, across all participants for each stabilisation method. **(C)** Weighted UniFrac distances between replicate species profiles derived from the **MCP**, by participant for each stabilisation method. **(D)** Aggregated weighted UniFrac distances between replicate species profiles derived from the **MCP**, across all participants for each stabilisation method. * = FDR P-value < 0.05 compared to frozen samples. Significance was assessed by linear mixed effect regression (LMER).


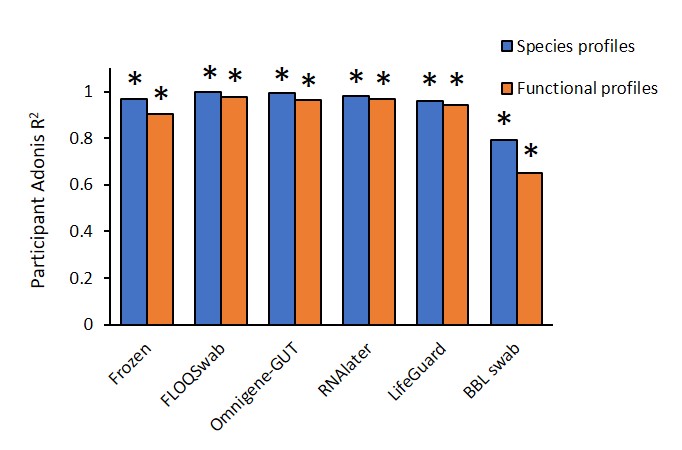


**Supplementary Figure S3.** Effect size from Adonis PERMANOVA analyses of Bray-Curtis dissimilarities between replicates of species (MCP) and functional profiles, for each stabilisation method. The participant R^2^ represents the amount of variance between replicates that can be explained by the participant. Permutations were set to 10^4^. P-values indicate significance of the variance explained by participant (R^2^). * = P-value = 1 x 10^-4^.


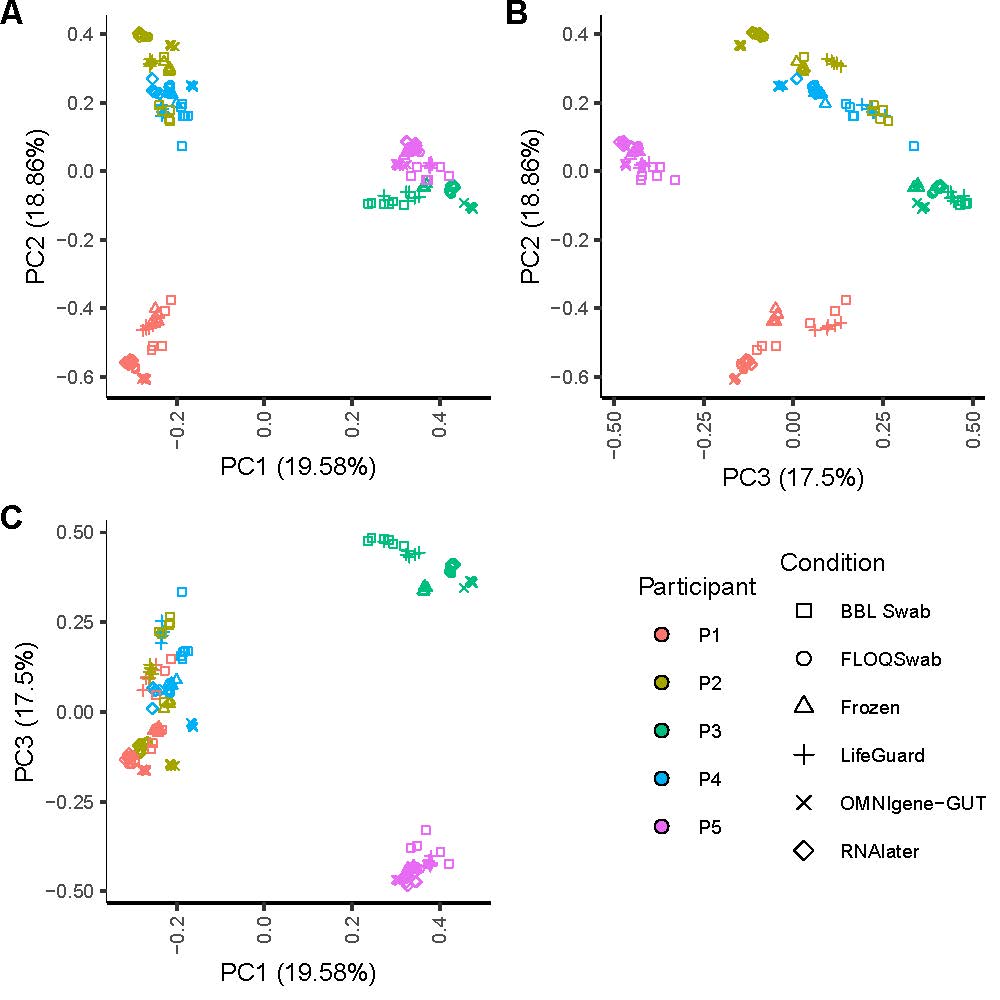


**Supplementary Figure S4.** Principal component analysis plot of Hellinger transformed species profiles (MCP) for the 180 samples, by participant (P1 to P5; varying colours) and storage method (varying shapes).


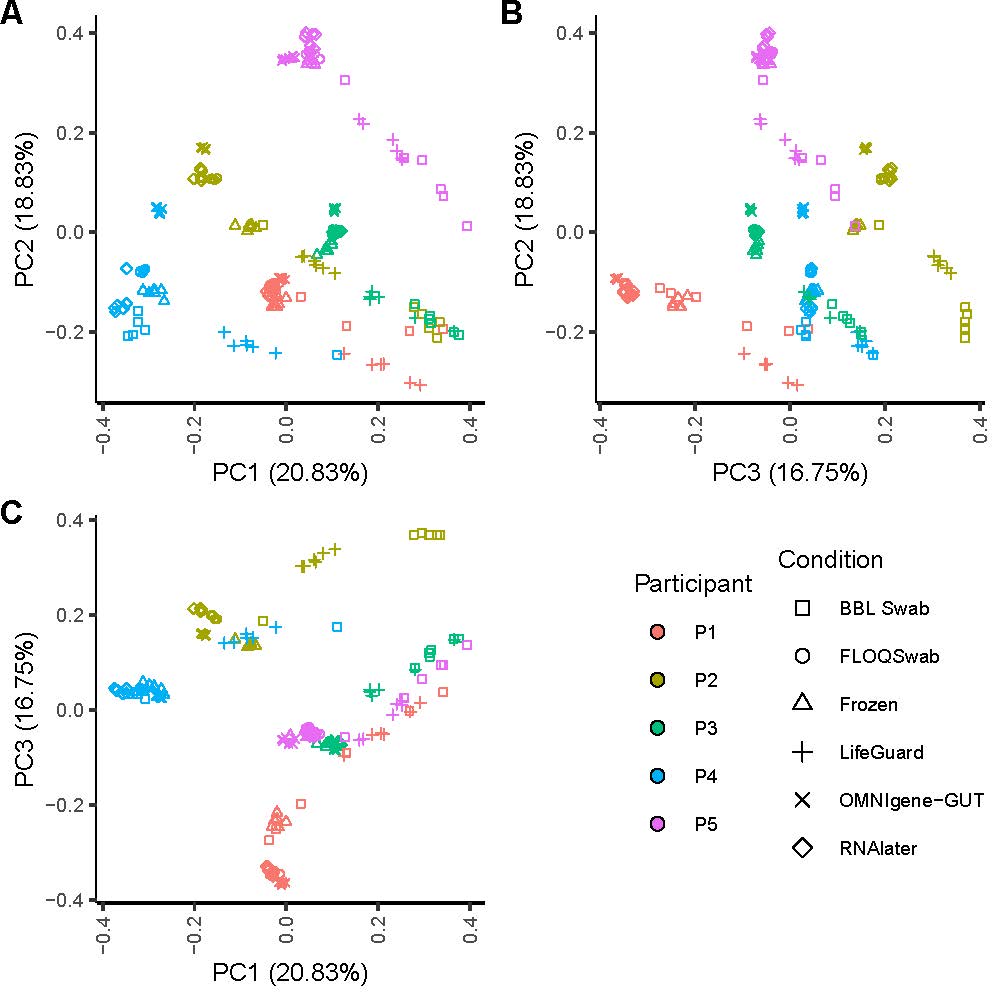


**Supplementary Figure S5:** Principal component analysis plot of Hellinger transformed species profiles (MetaPhlan3) for the 180 samples, by participant (P1 to P5; varying colours) and storage method (varying shapes).

**A**


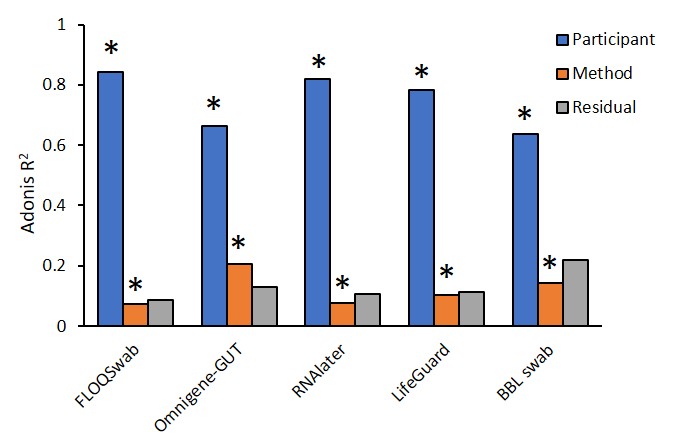


**B**


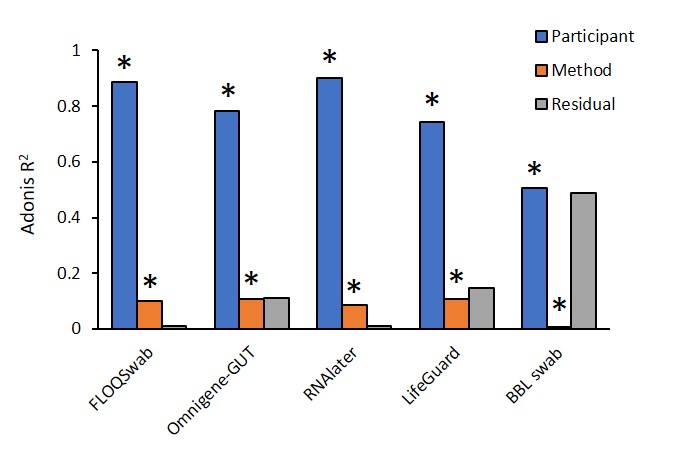


**Supplementary Figure S6.** Effect size from Adonis PERMANOVA analyses of Bray-Curtis dissimilarities for species (MCP) and functional profiles, comparing each stabilisation method to the frozen control. **(A)** Species profiles. **(B)** Functional profiles. The Participant R^2^ represents the amount of variance between replicates that can be explained by the participant. The Method R^2^ represents the amount of consistent variance between replicates that can be explained by the stabilisation method. The Residual R^2^ refers to the amount of unexplained variance. Permutations were set to 10^4^. P-values indicate significance of the variance explained by participant or method (R^2^). * = P-value = 1 x 10^-4^.


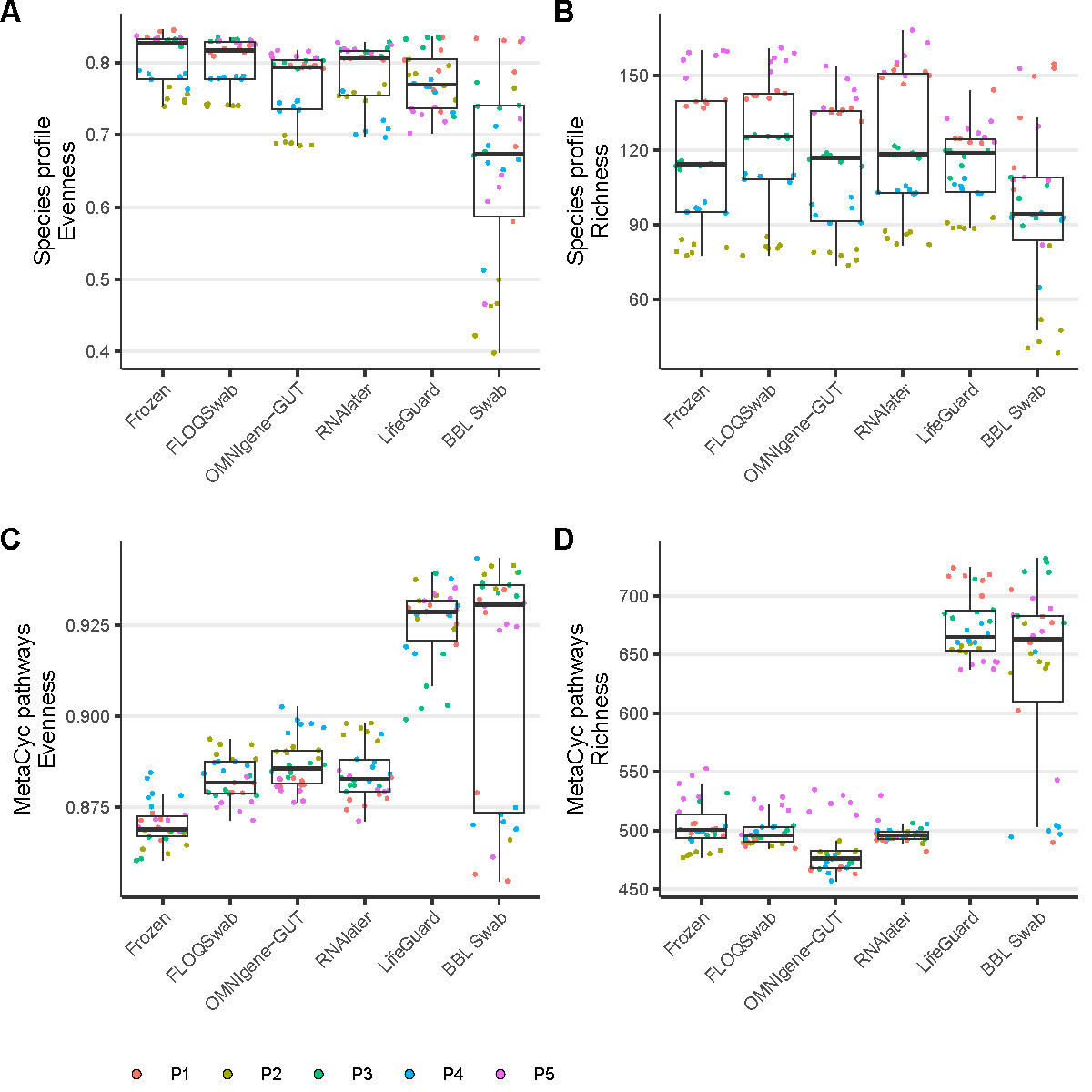


*

*

*

*

*

*

*

*

*

*

*

*

*

*

*

*

*

*

**Supplementary Figure S7.** Richness and Evenness of species (MCP) and functional profiles compared to frozen samples for each RT stabilisation method. **(A)** Species evenness across all participants for each stabilisation method. **(B)** Species richness across all participants for each stabilisation method. **(C)** Functional evenness across all participants for each stabilisation method. **(D)** Functional richness across all participants for each stabilisation method. Different colours represent different participants. * = FDR P-value < 0.05 compared to frozen samples. Significance was assessed by linear mixed effect regression (LMER).


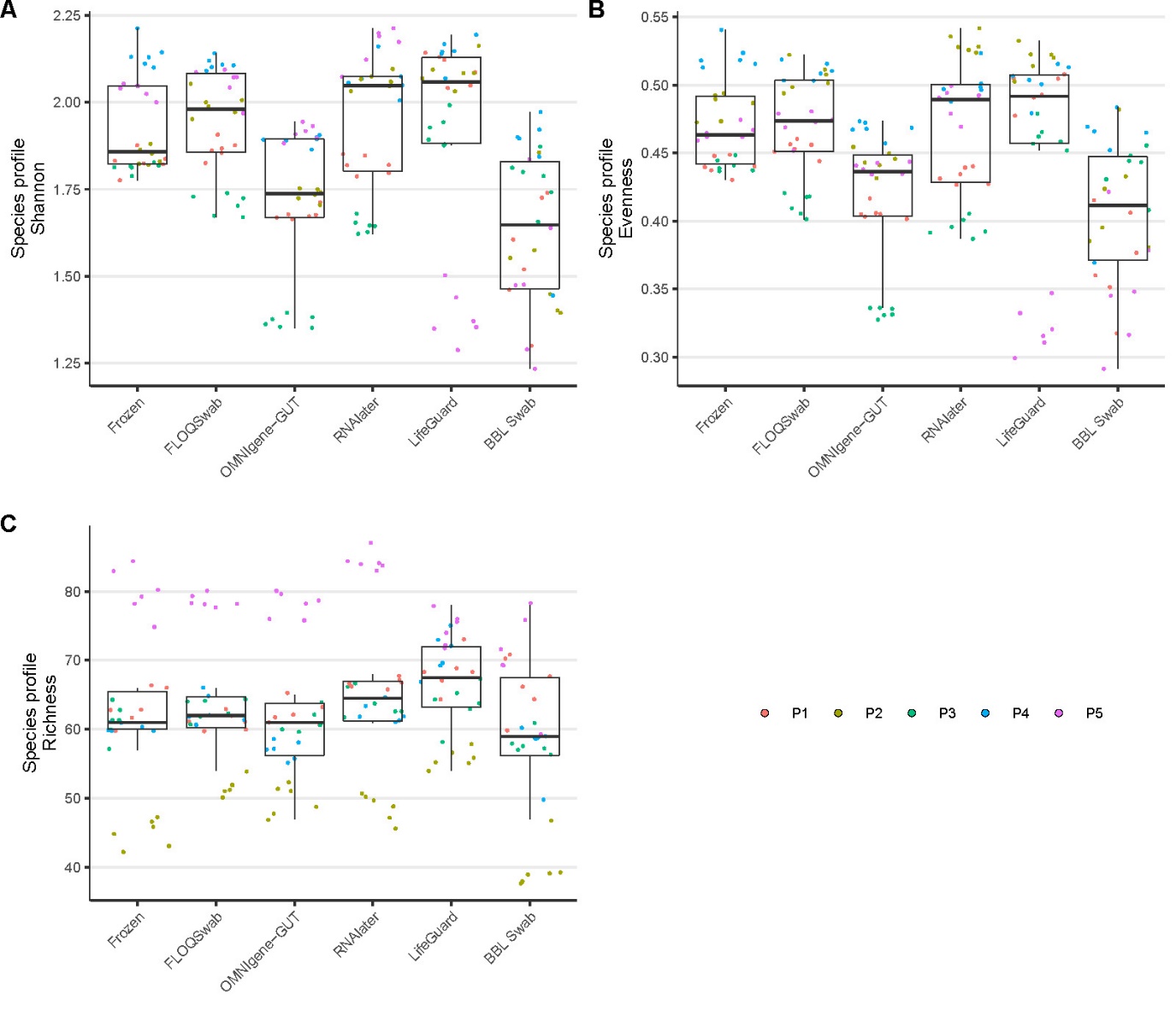


*

*

*

*

*

*

*

*

**Supplementary Figure S8.** Shannon diversity, richness and evenness of species profiles derived using MetaPhlan3 for each RT stabilisation method. **(A)** Shannon diversity across all participants for each stabilisation method. **(B)** Species evenness across all participants for each stabilisation method. **(C)** Species richness across all participants for each stabilisation method. Different colours represent different participants. * = FDR P-value < 0.05 compared to frozen samples. Significance was assessed by linear mixed effect regression (LMER).


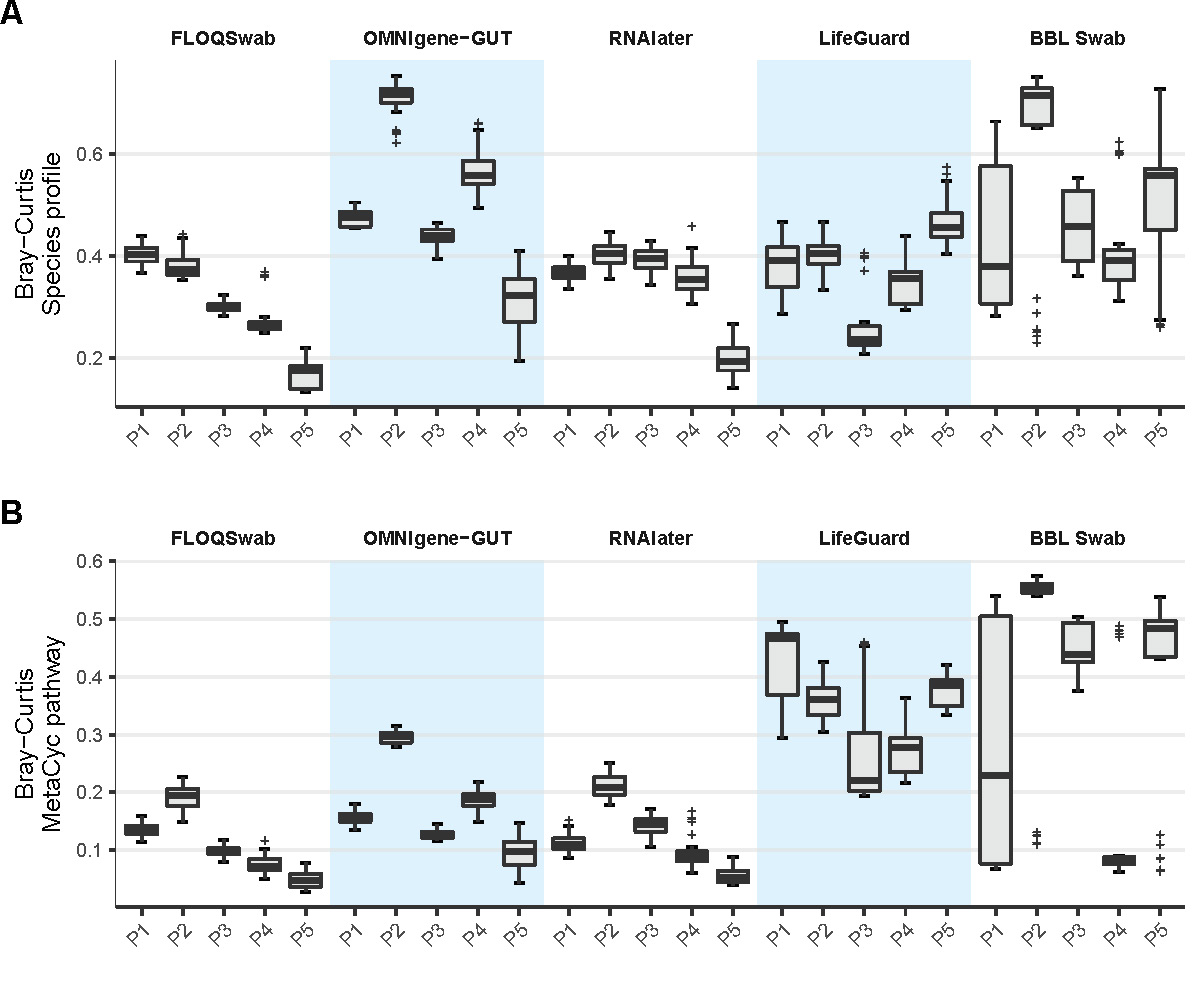


**Supplementary Figure S9.** Compositional reproducibility of species (MCP) and functional profiles by participant and stabilisation method. **(A)** Bray-Curtis dissimilarity of species profiles compared to flash-frozen profiles for each participant and stabilisation method. **(B)** Bray-Curtis dissimilarity of functional profiles compared to flash-frozen profiles for each participant and stabilisation method.


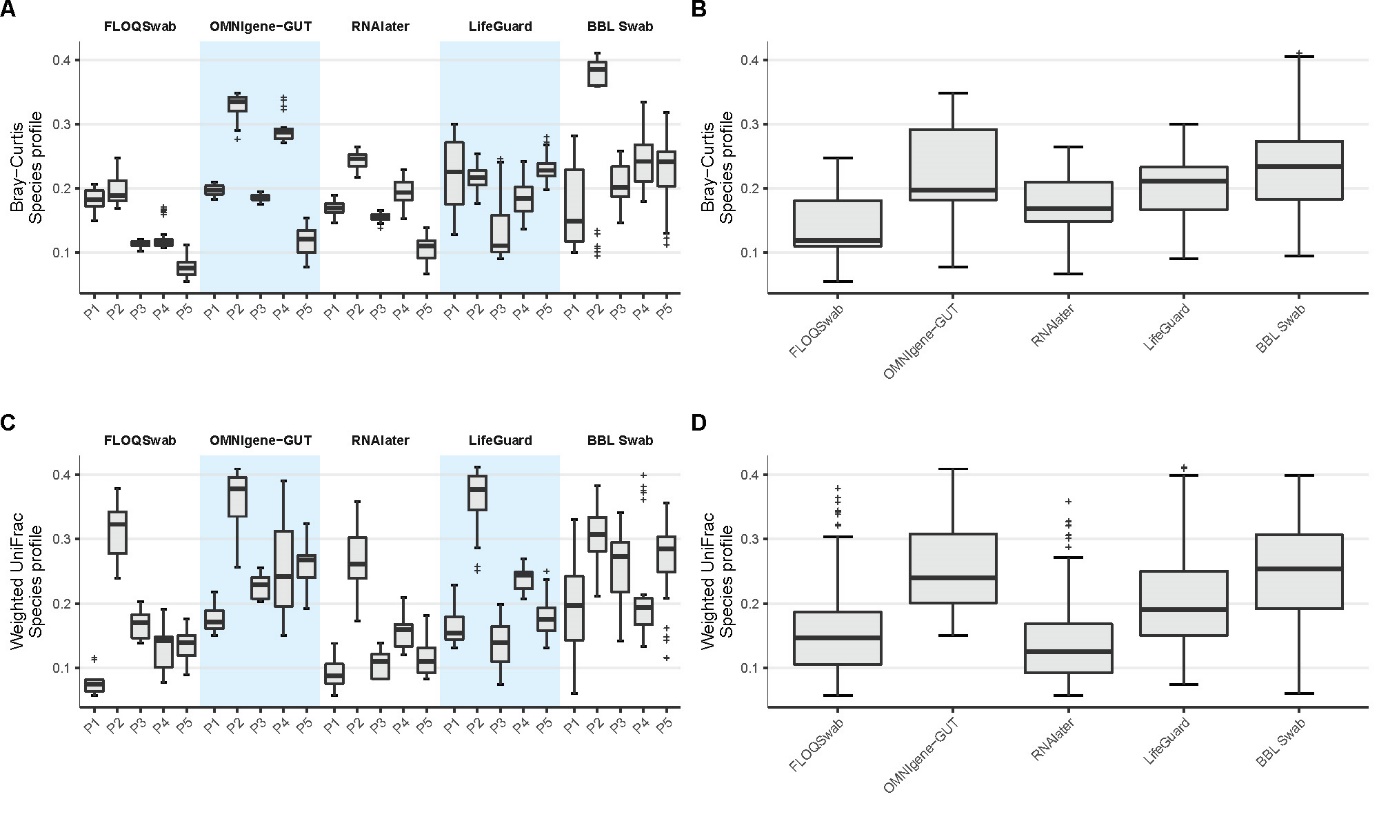


**a**

**a**

**b**

**c**

**d**

**b**

**b**

**c**

**d**

**b**

**Supplementary Figure S10.** Compositional reproducibility of aggregated species profiles for each stabilisation method. **(A)** Bray-Curtis dissimilarity of species profiles (**MetaPhlan3**) compared to flash frozen profiles, by participant for each stabilisation method. **(B)** Aggregated Bray-Curtis dissimilarity of species profiles (**MetaPhlan3**) compared to flash frozen profiles, across all participants for each stabilisation method. **(C)** W-UniFrac distances of species profiles (**MCP**) compared to flash frozen profiles, by participant for each stabilisation method. **(D)** Aggregated W- UniFrac distances of species profiles (**MCP**) compared to flash frozen profiles, across all participants for each stabilisation method. Letters: Boxes that do not share the same letter are significantly different at FDR P-value < 0.05. Statistical significance was assessed by LMER.


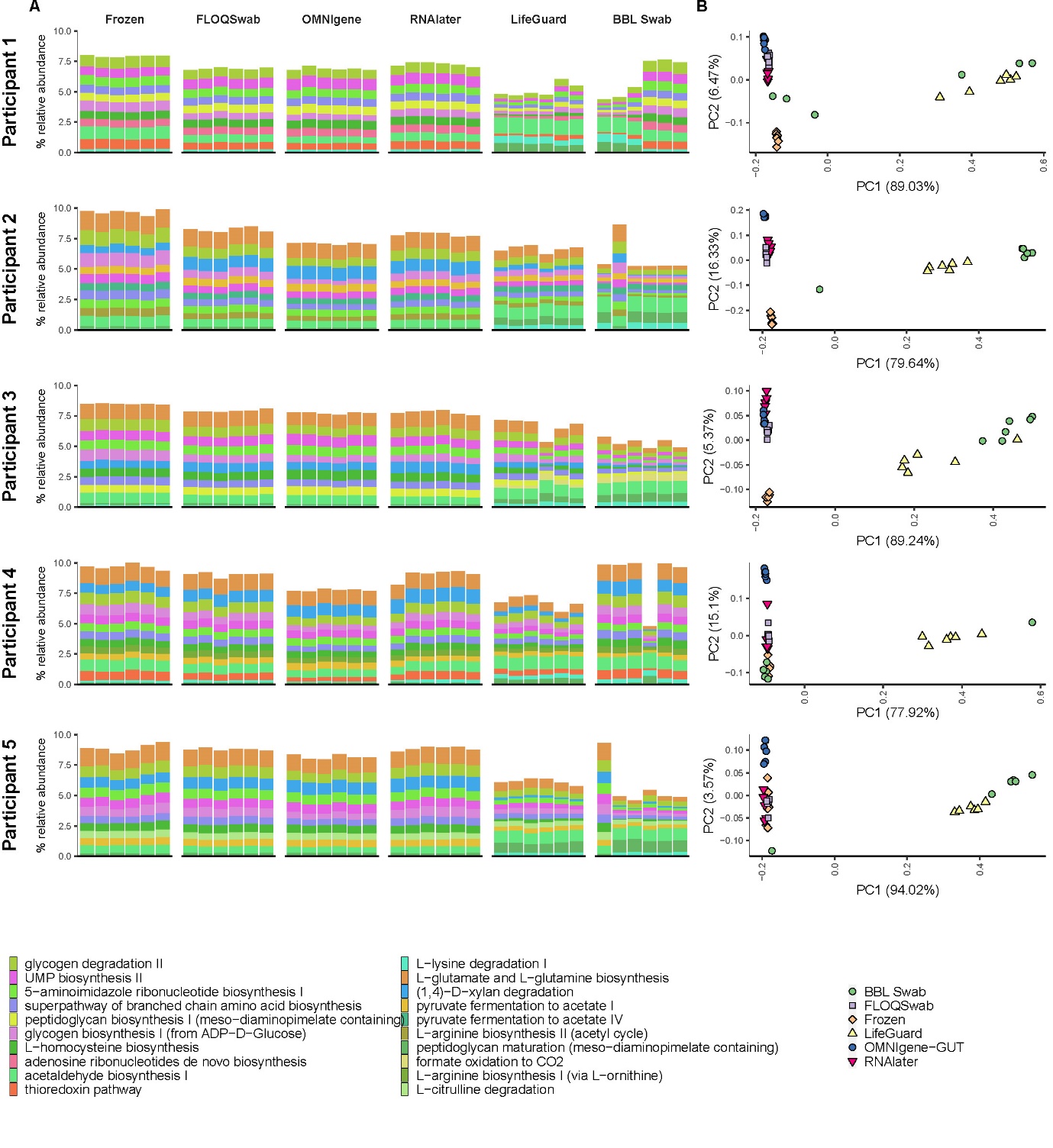


**Supplementary Figure S11**. Comparison of functional profiles for each replicate from the five participants. Profiles are organized by participant and stabilisation method. **(A)** Bar chart of the 10 non-redundant functional pathways with the highest mean abundance for each participant. **(B)** Principal component analysis plots of Hellinger transformed functional profiles are provided for each participant with each stabilisation method depicted by a different colour and shape combination.


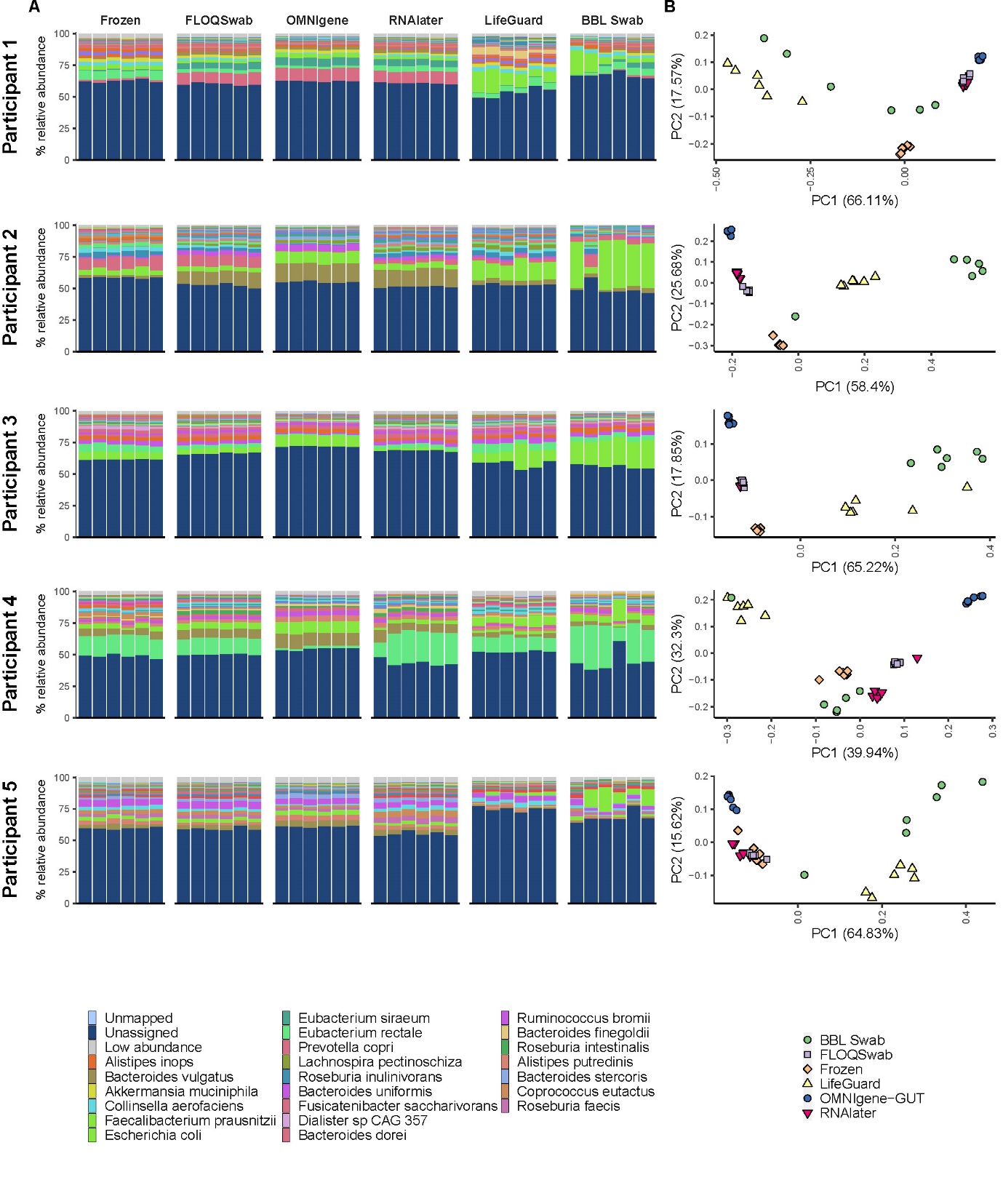


**Supplementary Figure S12.** Comparison of species profiles derived from **MetaPhlan3** for each replicate from the five participants. Profiles are organized by participant and stabilisation method. **(A)** Bar chart of the eight species with the highest mean abundance for each participant. The light and dark blue bars at the bottom of each bar plot indicate the percentage of unmapped and unassigned reads, respectively. The light grey bar at the top of each bar plot indicates the proportion of species with a minimum abundance <0.5% across all samples from a participant. **(B)** Principal component analysis plots of Hellinger transformed species profiles are provided for each participant with each stabilisation method depicted by a different colour and shape combination.


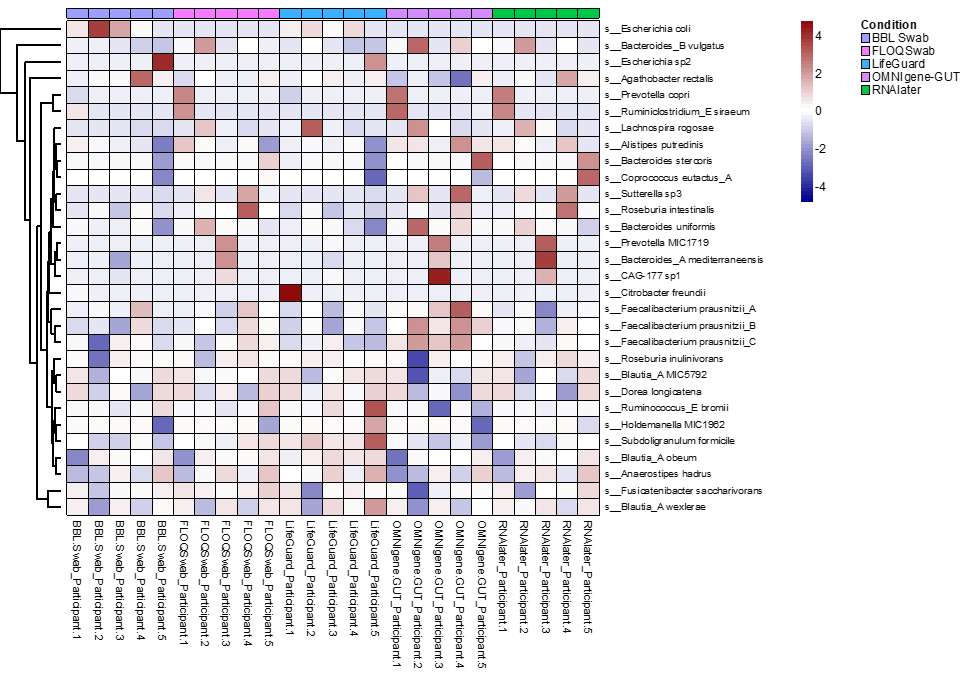


**Supplementary Figure S13**. Heatmap of the top 30 most variable species when comparing each stabilisation method to frozen profiles. Colours indicate differences compared to frozen profiles in mean centre-log ratio transformed relative abundances. For each participant, mean centre-log ratio transformed relative abundances of frozen profiles were subtracted from mean centre-log ratio transformed relative abundances of profiles for each stabilisation method.


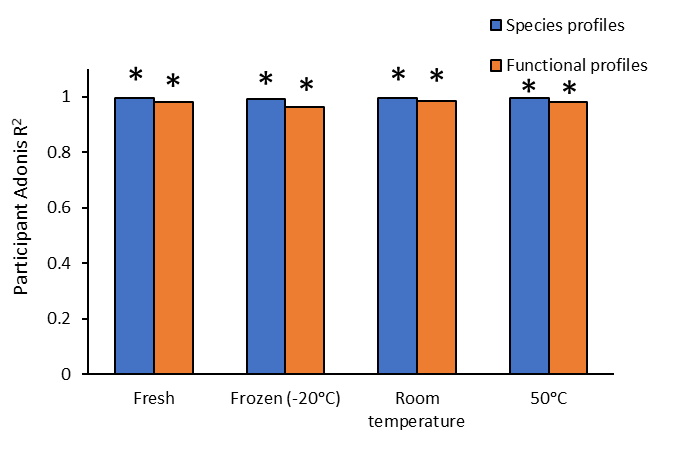


**Supplementary Fig S14.** Effect size from Adonis PERMANOVA analyses of Bray-Curtis dissimilarities of species and functional profiles between replicates of the FLOQSwab-ADT for each temperature treatment. The participant R^2^ represents the amount of variance between replicates that can be explained by the participant. Permutations were set to 10^4^. P-values indicate significance of the variance explained by participant (R^2^). * = P-value = 1 x 10^-4^.


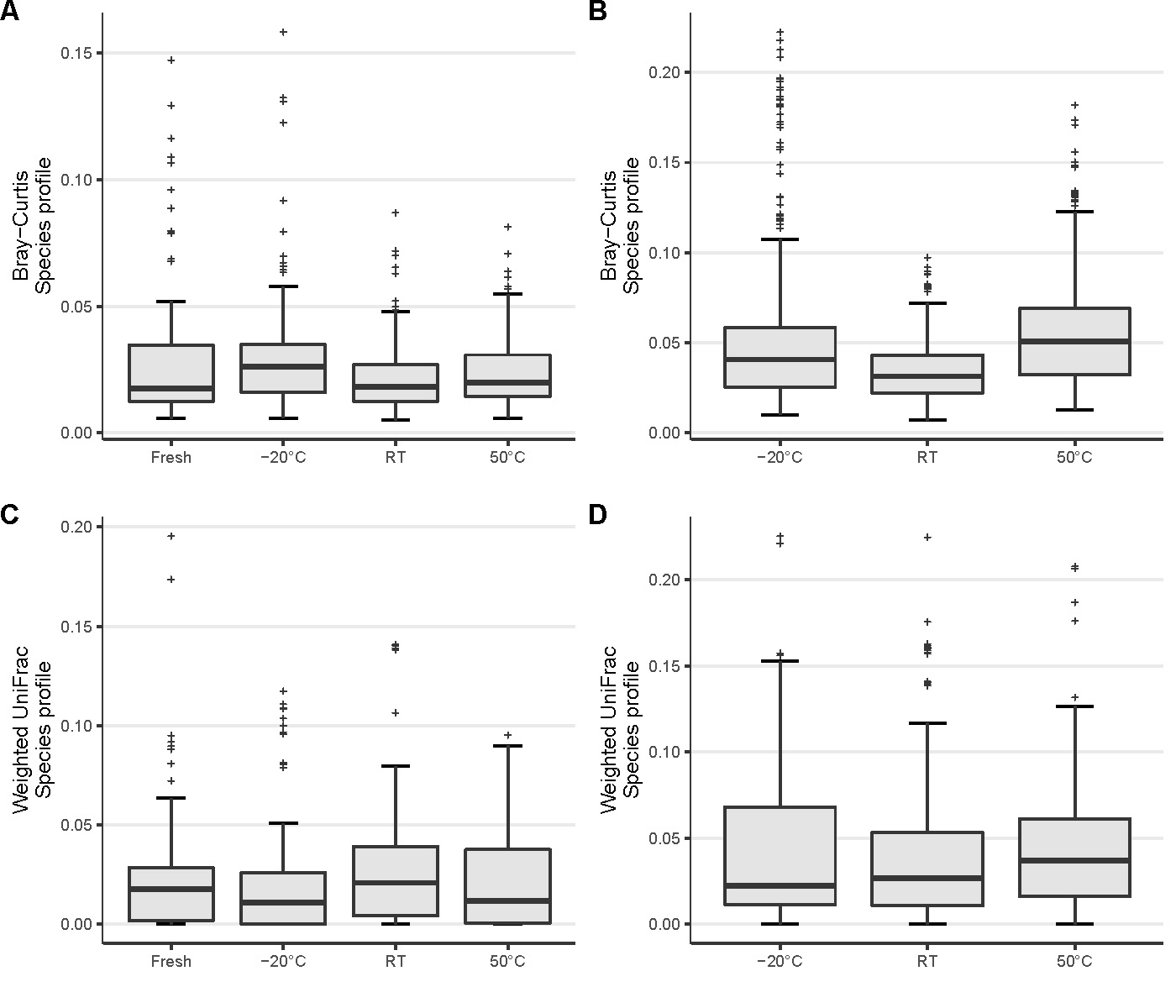


**b**

**a**

**a**

**a**

**a**

**a**

**Supplementary Figure S15**. Technical and compositional reproducibility for each temperature treatment compared to fresh controls. **(A)** Technical reproducibility assessed using aggregated Bray-Curtis dissimilarity of replicate species profiles derived from **MetaPhlan3** for all participants, at each temperature treatment. **(B)** Compositional reproducibility assessed using aggregated Bray-Curtis dissimilarity of species profiles derived from **MetaPhlan3** for all participants, at each temperature treatment compared to fresh controls. **(C)** Technical reproducibility assessed using aggregated W- UniFrac distances of species profiles derived from the **MCP** for all participants at each temperature treatment. **(D)** Compositional reproducibility assessed using aggregated W-UniFrac distances of species profiles derived from the **MCP** for all participants at each temperature treatment compared to fresh controls. Letters: Boxes that do not share the same letter are significantly different at FDR P-value < 0.05. Statistical significance was assessed by LMER.

**c**

**a**

**b**


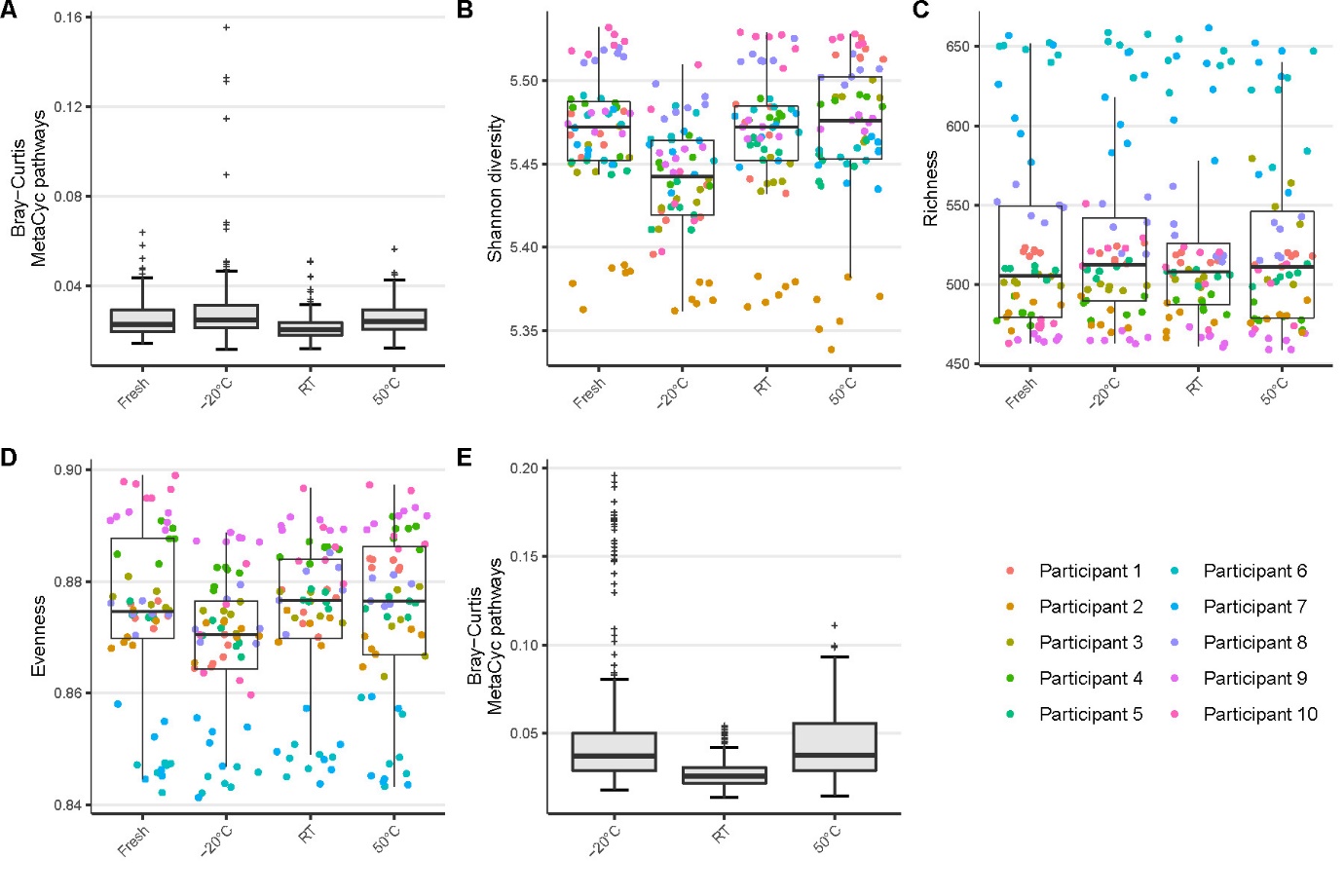


*

*

*

*

**a**

**b**

**c**

**Supplementary Figure S16.** Technical and compositional reproducibility of functional profiles for each temperature treatment compared to fresh controls. **(A)** Technical reproducibility assessed using aggregated Bray-Curtis dissimilarity of replicate functional profiles from all participants, at each temperature treatment. **(B)** Compositional reproducibility assessed using aggregated Shannon diversity of functional profiles for all participants at each temperature treatment. **(C)** Compositional reproducibility assessed using aggregated Richness of functional profiles for all participants at each temperature treatment **(D)** Compositional reproducibility assessed using aggregated Evenness of functional profiles for all participants at each temperature treatment **(E)** Compositional reproducibility assessed using aggregated Bray-Curtis dissimilarity of functional profiles for each temperature treatment compared to fresh controls. Different colours represent different participants. Gray crosses represent outliers. * = FDR P-value < 0.05 compared to fresh samples. Letters: Boxes that do not share the same letter are significantly different at FDR P-value < 0.05. Statistical significance was assessed by LMER.


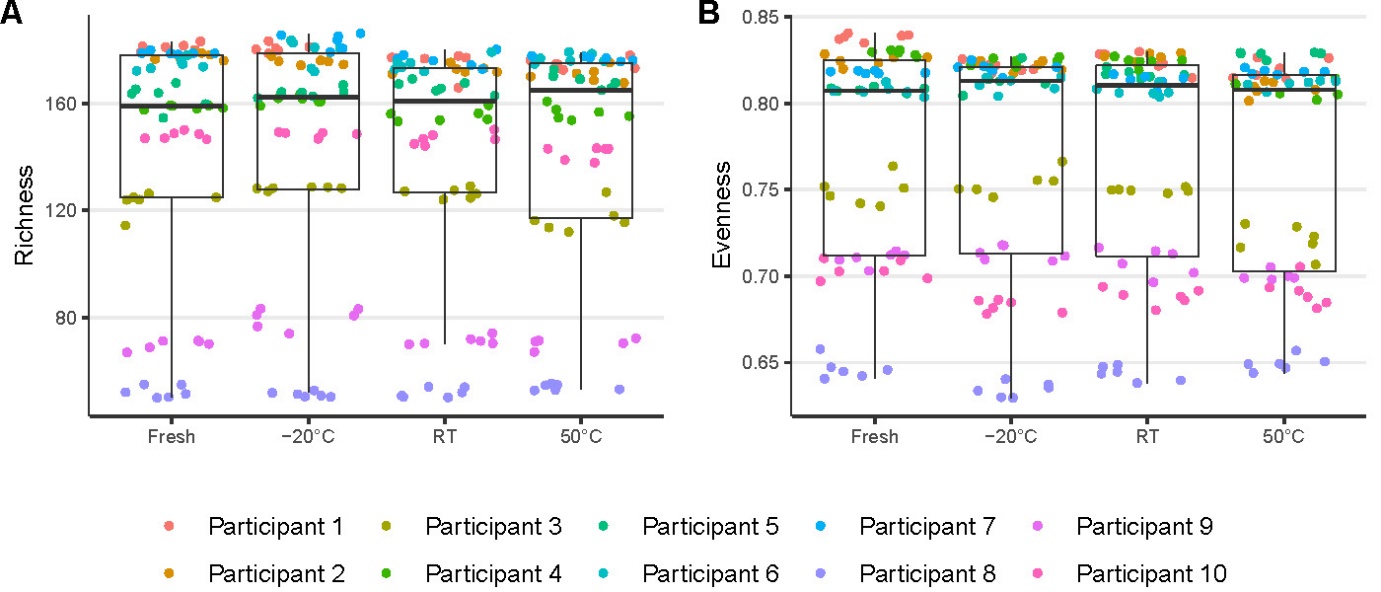


*

*

*

*

**Supplementary Figure S17**. Richness and evenness of species profiles (MCP) compared to fresh samples for each temperature treatment. **(A)** Species evenness across all participants for each temperature treatment. **(B)** Species richness across all participants for each temperature treatment. Different colours represent different participants. * = FDR P-value < 0.05 compared to fresh samples. Significance was assessed by linear mixed effect regression (LMER).


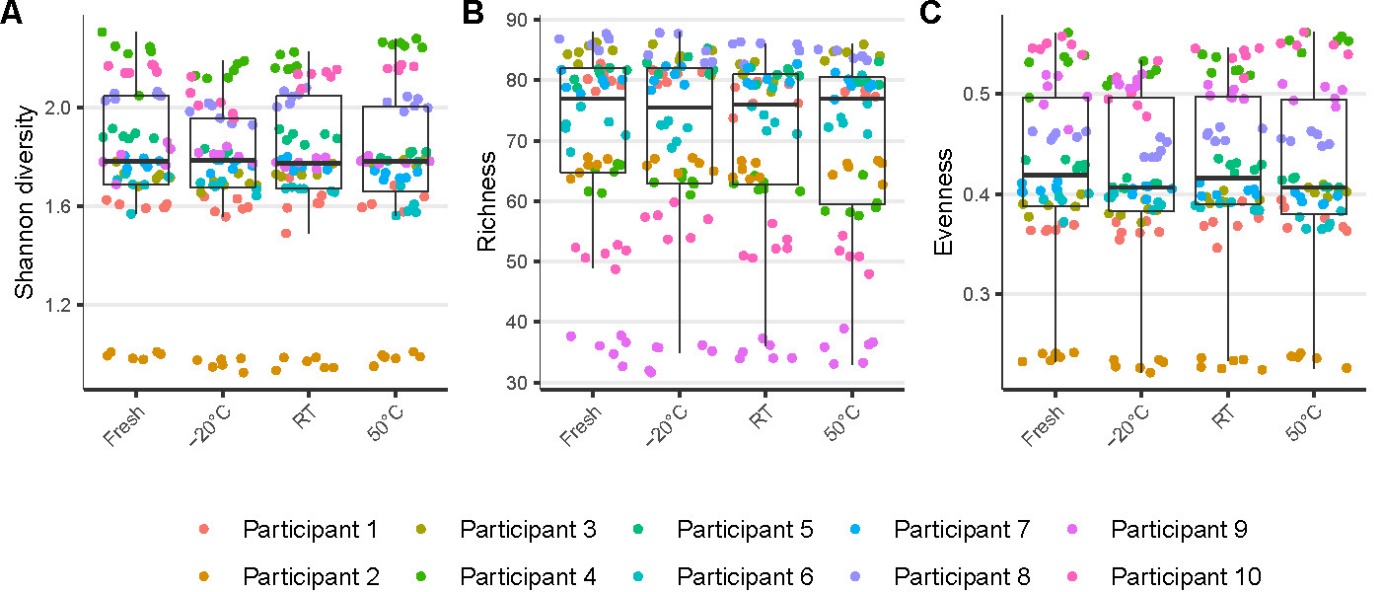


**Supplementary Figure S18.** Shannon diversity, richness and evenness of species profiles derived using **MetaPhlan3** for each temperature treatment compared to fresh samples. **(A)** Shannon diversity across all participants for each temperature treatment. **(B)** Species evenness across all participants for each temperature treatment. **(C)** Species richness across all participants for each temperature treatment. Different colours represent different participants. Significance was assessed by linear mixed effect regression (LMER).

**A**


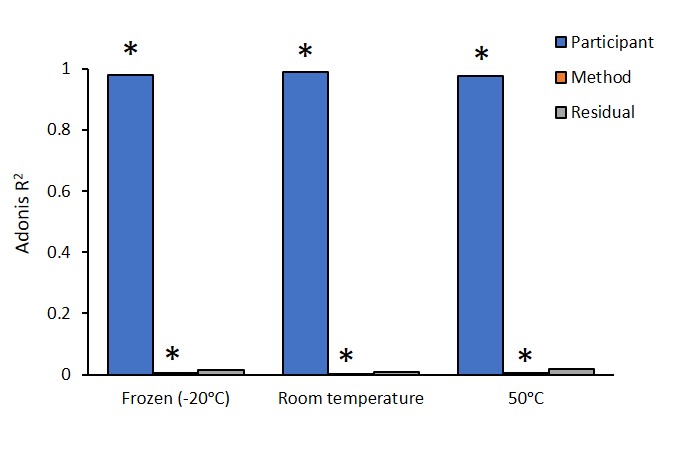


**B**


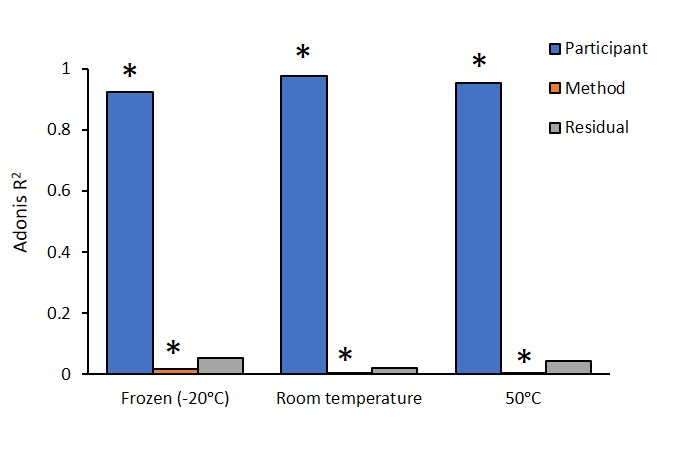


**Supplementary Figure S19.** Effect size from Adonis PERMANOVA analyses of Bray-Curtis dissimilarities for species (MCP) and functional profiles, comparing each temperature treatment to the fresh control. **(A)** Species profiles. **(B)** Functional profiles. The Participant R^2^ represents the amount of variance between replicates that can be explained by the participant. The Method R^2^ represents the amount of consistent variance between replicates that can be explained by the stabilisation method. The Residual R^2^ refers to the amount of unexplained variance. Permutations were set to 10^4^. P-values indicate significance of the variance explained by participant or method (R^2^). * = P-value = 1 x 10^-4^.


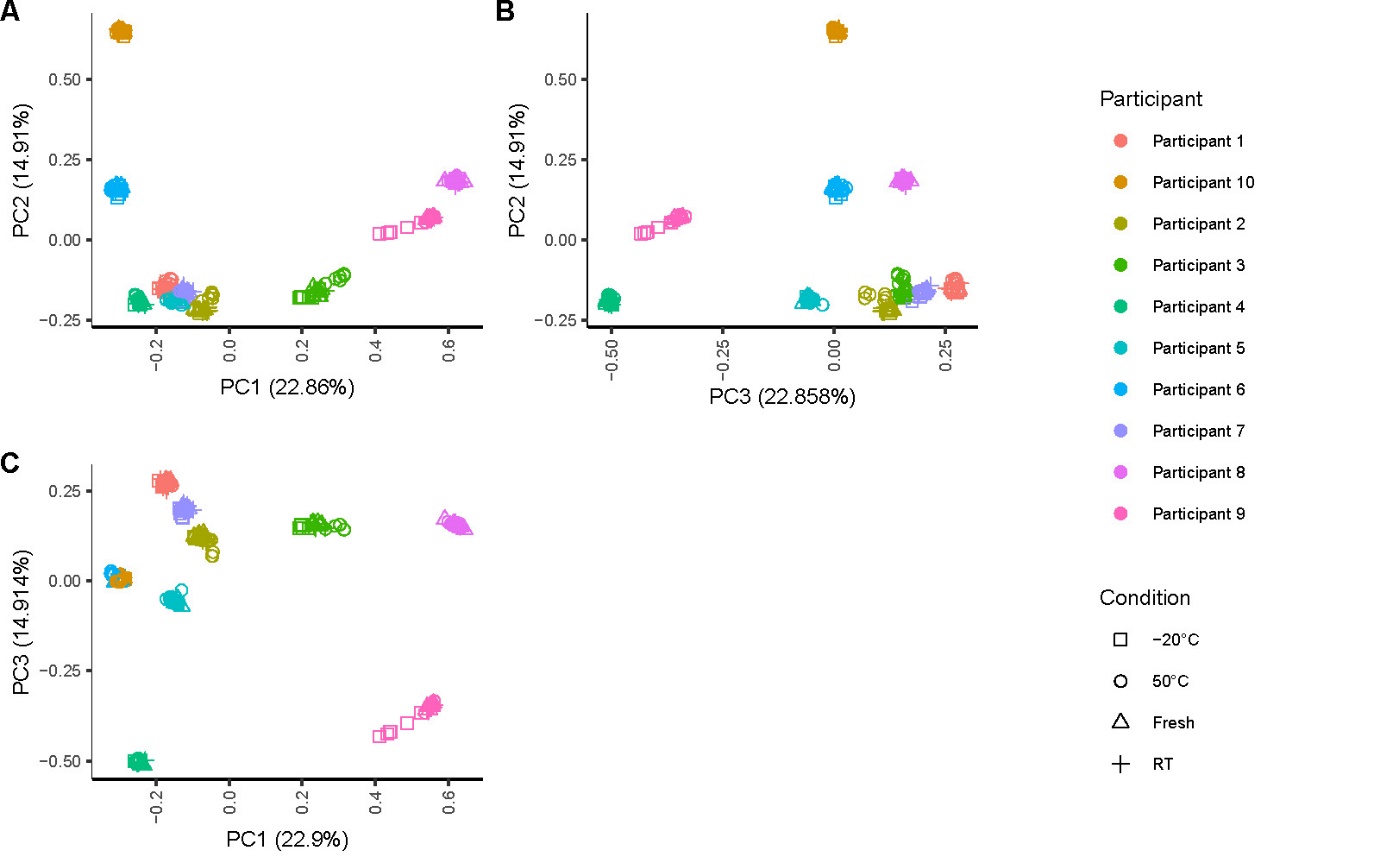


**Supplementary Figure S20.** Principal component analysis plot of Hellinger transformed species profiles derived from the **MCP** for the 239 samples, by participant.


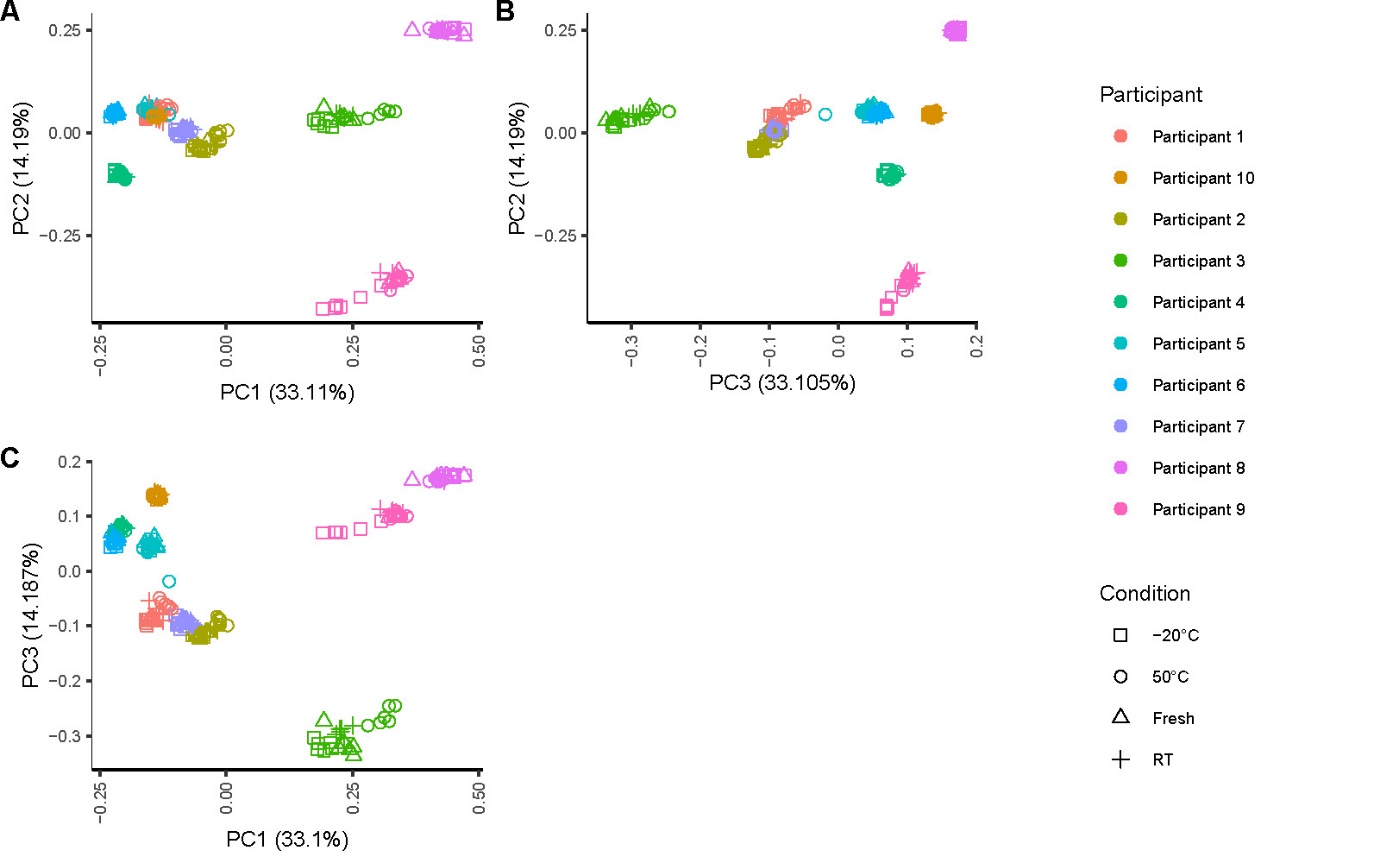


**Supplementary Figure S21.** Principal component analysis plot of Hellinger transformed species profiles derived from **MetaPhlan3** for the 239 samples, by participant.


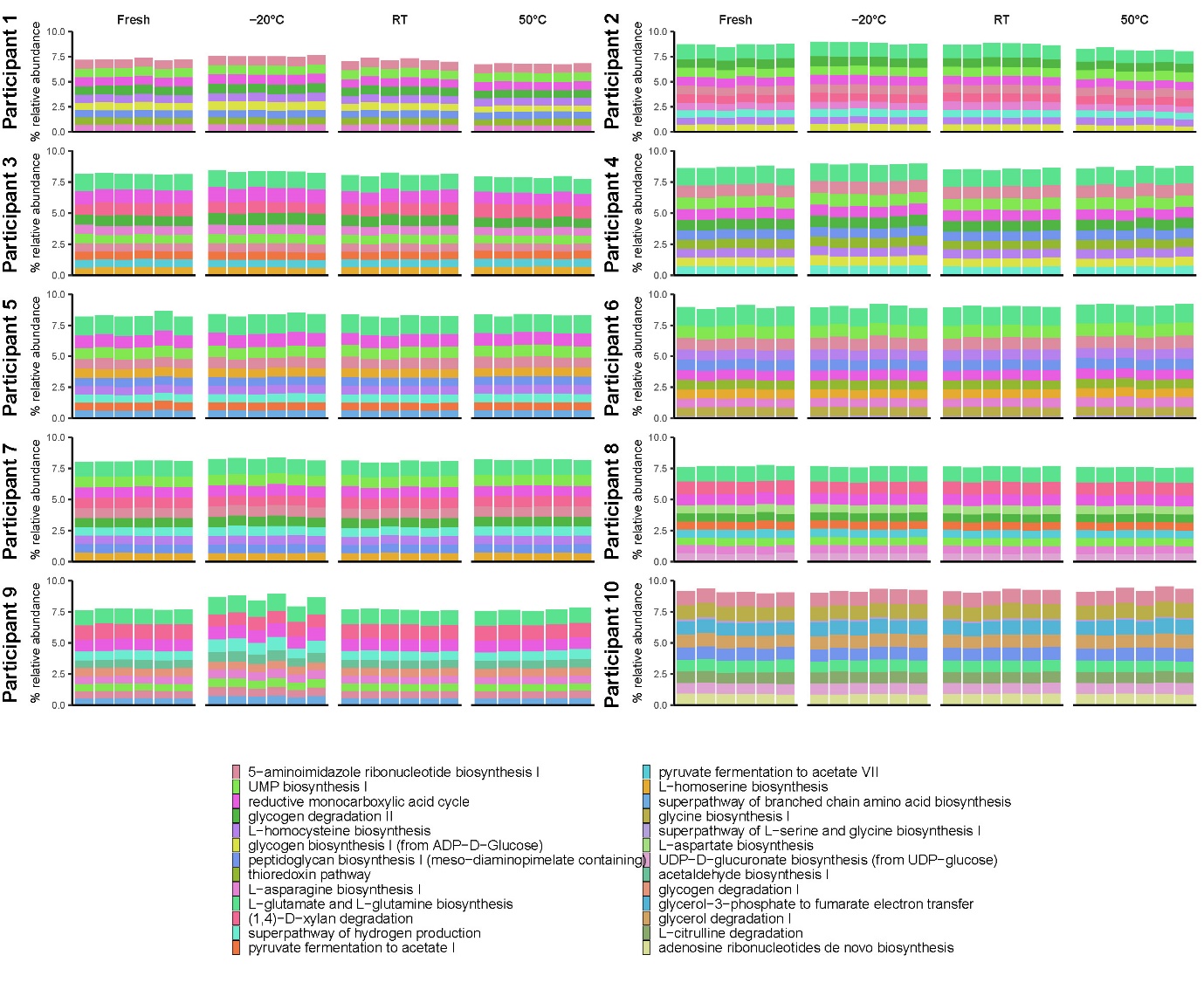


**Supplementary Figure S22**. Comparison of functional profiles for the 239 samples from the ten participants. Profiles are organized by participant and treatment method. The bar charts list the ten functional pathways with the highest mean abundance for each participant.


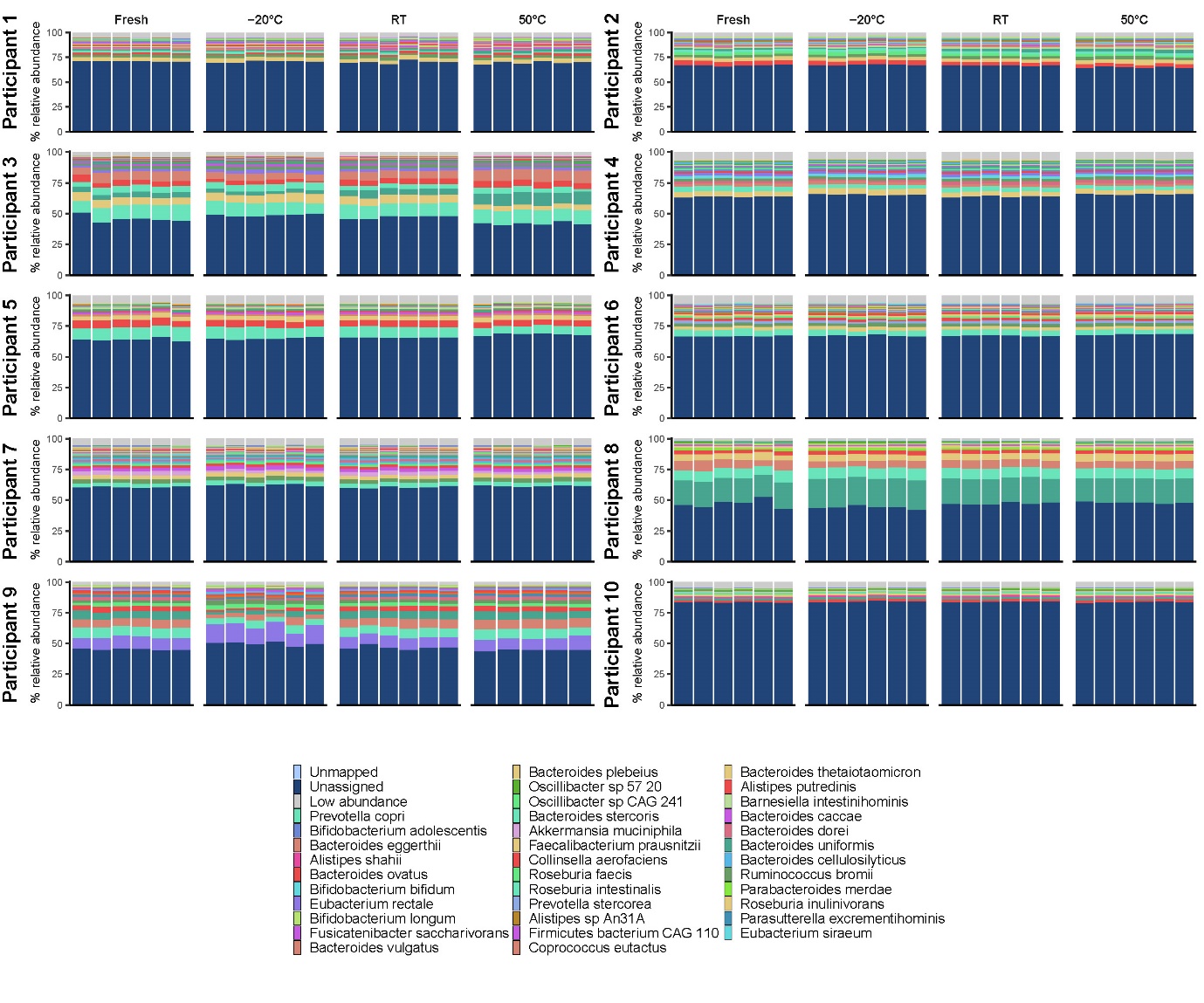


**Supplementary Fig S23.** Comparison of species profiles derived from **MetaPhlan3** for the 239 samples from the ten participants. Profiles are organized by participant and treatment method. The bar charts list the ten species with the highest mean abundance for each participant. The light and dark blue bars at the bottom of each bar plot indicate the percentage of unmapped and unassigned reads, respectively. The light grey bar at the top of each bar plot indicates the proportion of species with a minimum abundance <0.5% across all samples from a participant.
